# The Insufficient Activation of RIG-I–Like Signaling Pathway Contributes to Highly Efficient Replication of Porcine Picornaviruses in IBRS-2 Cells

**DOI:** 10.1016/j.mcpro.2021.100147

**Published:** 2021-09-14

**Authors:** Xiangle Zhang, Fan Yang, Kangli Li, Weijun Cao, Yi Ru, Shuying Chen, Shasha Li, Xiangtao Liu, Zixiang Zhu, Haixue Zheng

**Affiliations:** State Key Laboratory of Veterinary Etiological Biology, National Foot and Mouth Diseases Reference Laboratory, Key Laboratory of Animal Virology of Ministry of Agriculture, Lanzhou Veterinary Research Institute, Chinese Academy of Agricultural Sciences, Lanzhou, China

**Keywords:** senecavirus A, foot-and-mouth disease virus, RIG-I–like receptor signaling pathway, interferon, antiviral response, ACN, acetonitrile, CDS, coding sequence, CPE, cytopathic effect, DEP, differentially expressed protein, DMEM, Dulbecco's modified Eagle's medium, FBS, fetal bovine serum, FDR, false discovery rate, FMDV, foot-and-mouth disease virus, GO, Gene Ontology, HA, hemagglutinin, hpi, hours post infection, IBRS-2, Instituto Biologico-Rim Suino-2, IFN, interferon, IL-6, interleukin-6, IRF3, IFN regulatory factor 3, IRF7, IFN regulatory factor 7, ISG, interferon-stimulated gene, iTRAQ, isobaric tag for relative and absolute quantitation, JAK-STAT, Janus kinase signal transducer and activator of transcription, KEGG, Kyoto Encyclopedia of Genes and Genomes, MAVS, mitochondrial antiviral-signaling protein, MDA5, melanoma differentiation–associated protein 5, MOI, multiplicity of infection, PK-15, porcine kidney-15 cells, qPCR, quantitative PCR, RIG-I, retinoic acid–inducible gene I, RLR, RIG-I–like receptor, SeV, Sendai virus, SVV, Seneca Valley virus, TBK1, TANK-binding kinase 1, TCID50, 50% tissue culture infective dose

## Abstract

Seneca Valley virus (SVV) or commonly known as senecavirus A, is one of the picornavirus that is associated with vesicular disease and neonatal mortality in swine herds. Our previous study found that SVV replicates extremely faster in porcine Instituto Biologico-Rim Suino-2 (IBRS-2) cells than that in porcine kidney-15 (PK-15) cells. However, the underlying mechanism remains unknown. In this study, we comprehensively compared the expression features between IBRS-2 cells and PK-15 cells in response to SVV infection by an unbiased high-throughput quantitative proteomic analysis. We found that the innate immune response–related pathways were efficiently activated in PK-15 cells but not in IBRS-2 cells during SVV infection. A large amount of interferon (IFN)-stimulated genes were induced in PK-15 cells. In contrast, no IFN-stimulated genes were induced in IBRS-2 cells. Besides, we determined similar results in the two cell lines infected by another porcine picornavirus foot-and-mouth disease virus. Further study demonstrated that the Janus kinase signal transducer and activator of transcription signaling pathway was functioning properly in both IBRS-2 and PK-15 cells. A systematic screening study revealed that the aberrant signal transduction from TANK-binding kinase 1 to IFN regulatory factor 3 in the retinoic acid–inducible gene I–like receptor signaling pathway in IBRS-2 cells was the fundamental cause of the different innate immune response manifestation and different viral replication rate in the two cell lines. Together, our findings determined the different features of IBRS-2 and PK-15 cell lines, which will help for clarification of the pathogenesis of SVV. Besides, identification of the underlying mechanisms will provide new targets and an insight for decreasing the viral clearance rate and probably improve the oncolytic effect by SVV in cancer cells.

## Highlights

• Divergent innate immune responses were triggered by SVV in IBRS-2 and PK-15 cells.
• SVV induced higher levels of type I IFN in PK-15 cells than in IBRS-2 cells.
• IBRS-2 cell line has an aberrant RLR pathway but an intact type I IFN pathway.
• TBK1-mediated antiviral signal transduction was dysfunctional in IBRS-2 cells.

## Graphical Abstract

**Figure:**
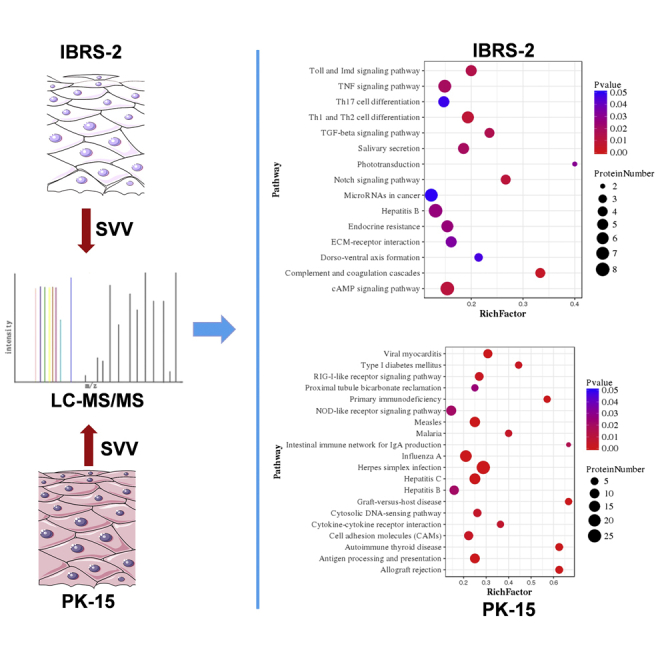

## In Brief

Both IBRS-2 and PK-15 cells have been widely used for porcine picornavirus research. However, the virus replicates faster and causes severer CPE in IBRS-2 cells than in PK-15 cells, and the underlying mechanism remains unknown. Proteomic analyses suggested that the RLR pathway was in a dysfunctional state in IBRS-2 cells. We finally determined that the disabled signal transduction from TBK1 to IRF3 in IBRS-2 cells was the fundamental cause of dysfunction of the RLR pathway during porcine picornavirus infection.

Seneca Valley virus (SVV), also known as senecavirus A, is a small and single-stranded RNA virus and the sole member of the genus *Senecavirus* within the family Picornaviridae. SVV is an oncolytic virus, which can propagate in human tumor cells, and has been used as an oncolytic virotherapy candidate in humans (1, 2). SVV infection also causes vesicular disease and neonatal mortality in swine (3). As an emerging picornavirus of swine, SVV has spread rapidly around the world since it was proved to be a causative agent in pigs in Canada in 2007 (4). The SVV cases have been reported constantly in the United States, China, and Brazil in recent years, causing significant economic losses, and it continues to be present in the swine herds in many countries in 2020 (5, 6, 7, 8) (https://umnswinenews.com/2020/02/28/senecavirus-a-continues-to-be-present-in-the-united-states-swine-herd/comment-page-1/; https://www.pig333.com/latest_swine_news/brazil-senecavirus-a-surveillance-increases-on-pig-farms_15849/). Although a lot of work has been done on investigation of SVV, there is still no available commercial vaccines and drugs against SVV, and many aspects of SVV infection characteristics, host range, and pathogenesis remain largely unknown, leaving the continuous spreading of the disease in many countries.

Cell lines have been used as important tools for studying virus–host interactions and physiological and pathophysiological processes during viral infection. It allows the examination of stepwise alterations in the structure and biology of host cell under viral infection and replication. SVV can replicate in many cell lines, including human-derived PER.C6, human embryonic kidney-293T, and H1299 cells (http://www.cfsph.iastate.edu/pdf/shic-factsheet-senecavirus-a, (9)), porcine-derived ST, SK-RST, porcine kidney-15 (PK-15), SK-6, and Instituto Biologico-Rim Suino-2 (IBRS-2) cells (http://www.cfsph.iastate.edu/pdf/shic-factsheet-senecavirus-a, (10)), as well as baby hamster kidney-21 cells (10, 11). Although all these cell lines are permissive to SVV amplification and can be used for SVV isolation, as an etiologic agent of pigs, the porcine cell lines have been widely used for studying of SVV (12, 13, 14, 15). Critical information on the pathogenesis of SVV infection, receptors used for viral entry, viral immune evasion mechanisms, and viral replication efficiency in porcine cells is especially valuable for establishing effective prevention and control strategies to counter this pathogen of great animal health concern.

Choosing the right cell line for specific experiments is key to getting the most reliable results. Therefore, a clear understanding of the context and properties of the selected cell line is critical for exploring biological mechanisms and predicting therapy response (16, 17). Our previous study found that different porcine cell lines reveal differential susceptibility to SVV (10). The comparison of growth kinetics of SVV in porcine PK-15 cells and IBRS-2 cells revealed that the IBRS-2 cell line was more permissible to SVV amplification than that in PK-15 cells, and SVV induced more significant cytopathic effect (CPE) in IBRS-2 cell line as well (10). The difference in sensitivity between PK-15 and IBRS-2 cells indicates a difference in susceptibility or proteomic profile of the two cell lines to SVV infection. IBRS-2 cell line might provide a better environment supportive of SVV replication. However, the discrepancies between the features of the two cell lines remain unclear.

To explore the potential mechanism that contributes to the different outcome of SVV replication in PK-15 and IBRS-2 cells, a high-throughput quantitative proteomic analysis of the proteome landscape and cellular responses of the two cell lines in response to SVV infection was performed and compared. Based on our analyses, we found that many interferon (IFN)-stimulated gene (ISG)–encoded proteins were highly upregulated in SVV-infected PK-15 cells. However, no ISG-encoded proteins were upregulated in IBRS-2 cells. Meanwhile, the Kyoto Encyclopedia of Genes and Genomes (KEGG) pathway enrichment analysis suggested that several differentially expressed proteins (DEPs) in PK-15 cells were enriched in retinoic acid–inducible gene I (RIG-I)–like receptor (RLR) signaling pathway, whereas no DEPs in IBRS-2 cells were enriched in this pathway. RLR pathway is essential for IFN production and ISG expression in response to RNA virus infection (18, 19). Therefore, we investigated and compared the state of RLR pathway activation in response to SVV infection. We found that SVV infection could not activate RLR signaling pathway in IBRS-2 cells, whereas it efficiently activated RLR signaling pathway in PK-15 cells. Similar features were observed in the two cell lines during another porcine picornavirus foot-and-mouth disease virus (FMDV) infection as well. We subsequently identified that the critical component of RLR signaling pathway TANK-binding kinase 1 (TBK1)–mediated signal transduction was abnormal in IBRS-2, resulting in insufficient type I IFN production and abrogated ISG expression. Our data suggested that the RLR signaling pathway in IBRS-2 cells was inactive during porcine picornavirus infection, and therefore, it cannot be used for exploring innate immune mechanisms during viral infections. In addition, the pig is very similar to humans with regard to anatomy, genetics, and physiology (20). It has been widely used as an animal model for human disease study (20, 21, 22), and it is also a prominent model for study of the oncolytic virotherapy of SVV. Therefore, clarification of the mechanism of viral replication characteristics of SVV in porcine cells will provide an insight for improving the oncolytic effect of SVV in cancer cells.

## Experimental Procedures

### Cells and Viruses

PK-15 and IBRS-2 cells were maintained in Dulbecco's modified Eagle's medium (DMEM; Gibco) supplemented with 10% fetal bovine serum (FBS; Biological Industries). All the cells were cultured at 37 °C under 5% CO_2._ A SVV strain CH-FJ-2017 (GenBank: KY74510) was isolated from pigs by our laboratory previously (10). FMDV type O strain O/BY/CHA/2010 (GenBank: JN998085) was isolated in China in 2010 and conserved by National Foot and Mouth Diseases Reference Laboratory, Lanzhou Veterinary Research Institute, Chinese Academy of Agricultural Sciences (23).

### Experimental Design and Statistical Rationale

PK-15 and IBRS-2 cells were mock infected (three control samples for each cell line) or infected (three viral-infected samples for each cell line) with SVV at a multiplicity of infection (MOI) of 0.5, respectively. The cells were harvested for quantitative proteomic analysis at 6 h postinfection (hpi). About 100 μg protein of each sample was then digested with Trypsin Gold (Promega). Three mock-infected PK-15 cell samples were labeled with isobaric tag for relative and absolute quantitation (iTRAQ) 116, 119, and 121 tags, respectively. Three SVV-infected PK-15 cell samples were labeled with iTRAQ 113, 114, and 115, respectively. The labeled peptide mixtures were pooled and dried *via* vacuum centrifugation. Similar treatment and iTRAQ labeling strategy was also conducted in IBRS-2 cells. In this study, we set SVV-1/Mock-1, SVV-1/Mock-2, SVV-1/Mock-3, SVV-2/Mock-1, SVV-2/Mock-2, SVV-2/Mock-3, SVV-3/Mock-1, SVV-3/Mock-2, SVV-3/Mock-3, SVV-3/SVV-2, SVV-3/SVV-1, SVV-2/SVV-1, Mock-3/Mock-2, Mock-3/Mock-1, and Mock-2/Mock-1 as comparison strategy for both PK-15 and IBRS-2 samples. The DEP fold-change cutoff value was set as 1.2-fold change (mean value of all comparison groups) and *p* value (*t* test of all comparison groups) less than 0.05. The DEPs were screened by volcano plot analysis, which is detected by Student's *t* test. The CV, which is defined as the ratio of the SD to the mean, was employed to assess the reproducibility. A lower CV value indicates better reproducibility.

### Plasmids

The coding sequence (CDS) fragments of porcine RIG-I (GenBank: EU126659), melanoma differentiation–associated protein 5 (MDA5) (GenBank: EU006039), mitochondrial antiviral-signaling protein (MAVS) (GenBank: EU082069), TBK1 (GenBank: EU091339), IFN regulatory factor 3 (IRF3) (GenBank: KC860781), and IFN regulatory factor 7 (IRF7) (GenBank: EU294309) with an additional hemagglutinin (HA) tag at the C terminus of each CDS were obtained using the gene synthesis method and cloned into pcDNATM3.1/myc-His(-)A vector (Invitrogen) to yield the HA-tagged expression plasmids of these innate immune molecules. The CDS for HA tag was as follows: 5′-TACCCATACGACGTCCCAGACTACGCT-3′. All the constructed expressing plasmids were analyzed and verified by DNA sequencing. The porcine IFN-β–promoter-luciferase reporter plasmid was generated by our laboratory previously (24). The pRL-TK Renilla luciferase reporter plasmid was kindly provided by Professor Hongbing Shu (Wuhan University, China) (25).

### Antibodies and Reagents

The commercial antibodies used in this study include anti-KIF11 mouse antibody (Santa Cruz Biotechnology), anti-MX1 rabbit antibody (Sigma), anti-IFIT1 mouse antibody (Sigma), anti-GSDMA rabbit antibody (Abcam), anti-MAP4K4 rabbit antibody (Abcam), anti-DEPD5 rabbit antibody (Thermo Fisher Scientific), anti-TRIB1 rabbit antibody (Abcam), anti-ZNF384 rabbit antibody (Thermo Fisher Scientific), anti-UBC13 mouse antibody (Santa Cruz Biotechnology), anti-Cystatin C mouse antibody (Novus Biologicals), anti-IGFBP2 mouse antibody (Santa Cruz Biotechnology), and anti-β-actin mouse antibody (Santa Cruz Biotechnology). Lipofectamine 2000 Transfection Reagent (Thermo Fisher Scientific) was used for transfection of plasmids. Porcine IFN-α and IFN-β were prepared by our laboratory previously (unpublished data).

### Viral Infection

Monolayers PK-15 or IBRS-2 cells were washed with PBS for three times and then challenged with SVV or FMDV at the indicated MOI at 37 °C. The unabsorbed viruses were removed by washing with PBS after 1 h adsorption, and the cells were then maintained in DMEM supplemented with 1% FBS at 37 °C for appropriate time. The mock-infected cells were subjected to similar treatment in parallel as mentioned previously, using the basic medium for incubation, and collected at appropriate time. The virus titers or viral replication state were evaluated for subsequent analysis.

### Sample Preparation, Trypsin Digestion, and iTRAQ Labeling

IBRS-2 and PK-15 cells were mock infected or infected with SVV at an MOI of 0.5, and the cells were then collected at 6 hpi and lysed in ice-cold lysis buffer containing different kinds of protease inhibitors for 15 min on ice as described previously (26). The cell lysates were sonicated on ice and centrifuged at 4 °C, 25,000*g* for 20 min to remove the cellular debris. The concentration of the protein in the resulting supernatant was quantified with the bicinchoninic protein assay reagent (Thermo Fisher Scientific), and the samples were used in proteomic detection. For tryptic digestion, 100 μg protein of each sample was digested with 10 μl trypsin (0.5 μg/μl) at 37 °C for 4 h. An additional digestion of 8 h was performed using equal volume of trypsin (trypsin-to-protein ratio of 1:1). The trypsin-digested peptides were collected by vacuum centrifugation and dissolved in 0.5 M triethylammonium bicarbonate (Sigma) and then labeled with different iTRAQ reagents (AB Sciex) for 2 h at room temperature following the manufacturer's protocol. The peptides from mock-infected IBRS-2 or PK-15 cells were labeled with iTRAQ 113 tag, and the peptides from SVV-infected IBRS-2 or PK-15 cells were labeled with iTRAQ 116 tag. For the biological replicates, the iTRAQ 114 and 115 tags were incubated with peptide samples obtained from another two batches of mock-infected IBRS-2 or PK-15 cells, respectively. The iTRAQ 119 and 121 tags were incubated with peptide samples from another two batches of SVV-infected IBRS-2 or PK-15 cells, respectively. After the labeling, the labeled samples from IBRS-2 cells were thoroughly mixed, desalted, and lyophilized to dryness through vacuum centrifugation. The peptides were desalted with a Strata X C18 column as described previously (27).

### Peptide Fractionation and Mass Spectrometric Analysis

Before mass spectrometric analysis, the iTRAQ-labeled peptides were separated using a Shimadzu LC-20AB HPLC Pump system as previously described (28). In brief, the peptides were reconstituted with 4 ml of buffer A (25 mM NaH_2_PO_4_ in 25% acetonitrile [ACN]) and loaded onto the column. The buffer A and a linear gradient of buffer B (5–35%, 25 mM NaH_2_PO_4_, 1 M KCl in 25% ACN) were then used for elution of the peptides and preparation of the fractions. The resulting 20 fractions (pooled by ∼80 eluted peptides) were collected and desalted by Strata X C18 column. Each fraction was then frozen, concentrated, and lyophilized to dryness.

Each fraction was resuspended with 2% ACN in 0.1% formic acid to reach a concentration of 0.5 μg/μl. The insoluble impurities were removed by centrifuge at 20,000*g* for 10 min. The samples were then loaded onto a C18 trap column using a LC-20AD nano-HPLC instrument (Shimadzu) by the autosampler. The C18 trap column was connected to a analytical C18 column (inner diameter of 75 μm, column diameter of 3.6 μm, and column length of 15 cm). The peptides were eluted and separated using a linear gradient of buffer C (5–80%, 98% ACN in 0.1% formic acid), connected to a Q-Exactive mass spectrometer (Thermo Fisher Scientific). MS data were acquired using the Xcalibur software (Thermo Fisher Scientific). The precursor scan was performed with a resolution of 70,000 in the range 350 to 1600 *m/z*, and the MS/MS spectra were acquired with a resolution of 17,500 in higher energy collisional dissociation mode at >100 *m/z*. The 20 most abundant precursor ions above a threshold ion count of 10,000 were selected from the precursor scan for MS/MS sequencing.

Protein database searching and protein identification from MS raw data were carried out using the Proteome Discoverer software, version 1.4 (Thermo Fisher Scientific) and Mascot search engine, version 2.3.02 (Matrix Science, Inc; http://www.matrixscience.com/) as described previously (27). The MS/MS spectra were searched against the Swiss-Prot *Sus scrofa* sequence database (the sequence database consisted of forward and reversed sequences; 49,792 entries searched; https://ftp.uniprot.org/pub/databases/uniprot/current_release/knowledgebase/reference_proteomes/Eukaryota/UP000008227/, downloaded December 2020). At least one unique peptide was necessary for the identified protein. For protein identification, one missing tryptic cleavage was permitted. Mass tolerances of 20 ppm and fragment mass tolerance of 0.05 Da was allowed. Oxidation (M) and iTRAQ8plex (Y) were set as potential variable modifications, and carbamidomethyl (C), iTRAQ8plex (N-term), as well as iTRAQ8plex (K) were considered as fixed modifications. The false discovery rate (FDR) was computed using N(decoy) ∗ 2/[N(decoy) + N(target)] as previously described (29).

The matched peptide spectrum after searching was filtered using the Proteome Discoverer software to make sure that the overall FDR was less than 0.01. The protein quantification for iTRAQ analysis was performed by IQuant, version 2.2.1 (BGI Group), and then a series of confident proteins were acquired based on the parsimony principle (30, 31). The protein level will be filtered again at an FDR of 1% (protein-level FDR ≤ 0.01) using the picked protein FDR strategy (32) to decrease the false-positive rate. Briefly, the main IQuant quantitation parameters were set as follows: Quant-peptide, use all unique peptide; Quant-number, at least one unique spectra; Normalization, VSN; Protein-Ratio, weighted average; Statistical Analysis, and permutation tests.

### Bioinformatics Analysis of Proteomics Data

The DEPs in the IBRS-2 or PK-15 cells in response to SVV infection were determined based on the quantification fold change >1.2 and the *p* value <0.05 (SVV-infected cells *versus* mock-infected cells) as described previously (28, 33, 34, 35). The subcellular localization analysis was performed by the WoLF PSORT database (http://www.genscript.com/psort/wolf_psort.html); the Gene Ontology (GO; http://geneontology.org/), Clusters of Orthologous Groups of proteins (http://www.ncbi.nlm.nih.gov/COG/), and KEGG (http://www.genome.jp/kegg/) databases were selected for subsequent bioinformatics analysis in this study. Significant proteins were functionally determined by annotation enrichment analysis using annotations from the UniProtKB keywords (36).

### Western Blotting

The mock-infected or virus-infected cells were collected at different hours postinfection and washed with PBS twice to remove cell debris and FBS. The cells were then lysed with ice-cold lysis buffer as described previously (26). The lysates were subsequently boiled with sample buffer (2% SDS, 10% glycerol, 60 mM Tris [pH 6.8], 5% β-mercaptoethanol, and 0.01% bromophenol blue) for 10 min to allow denaturation of the proteins and followed by centrifugation at 20,000*g* at 4 °C to remove debris. The supernatants were analyzed by SDS-PAGE and transferred onto polyvinylidene difluoride membranes (Millipore). About 5% skim milk and 0.5% Tween-20 in Tris-buffered saline was used as blocking buffer to prevent nonspecific binding of the antibodies to the polyvinylidene difluoride membranes. After 2 h blocking of the membranes, appropriate primary and secondary antibodies were incubated to form the protein–antibody complexes, and the complex was visualized by enhanced chemiluminescent substrate.

### Real-Time Quantitative PCR

Total cellular RNA was isolated using the TRIzol Reagent (Invitrogen) according to the manufacturer's protocol. The complementary DNA was synthesized using 2 μg of cellular RNA as template, and the M-MLV reverse transcriptase (Invitrogen) was used for reverse transcription. One-Step PrimeScript RT-PCR Kits (Perfect Real Time) (TaKaRa Bio) were used to evaluate the expression of cellular and viral mRNA, and the reaction was carried out using Mx3005P quantitative PCR (qPCR) System (Applied Biosystems). Porcine GAPDH was used as the housekeeping gene to normalize total RNA. Relative gene expression was quantified by calculating the 2^−△△CT^ (where CT is threshold cycle). All the used qPCR primers in this study are listed in Table 1.

**Table 1:** The qPCR primers used in this study

Target gene	Primers (5′-3′)	
*SVV*	Forward Reverse	AGAATTTGGAAGCCATGCTCT GAGCCAACATAGARACAGATTGC
*FMDV*	Forward Reverse	CACTGGTGACAGGCTAAGG CCCTTCTCAGATTCCGAGT
*GAPDH*	Forward Reverse	ACATGGCCTCCAAGGAGTAAGA GATCGAGTTGGGGCTGTGACT
*IFN-β*	Forward Reverse	GCTAACAAGTGCATCCTCCAAA AGCACATCATAGCTCATGGAAAGA
*MDA5*	Forward Reverse	TTCACGAGCGACCTCTGGAT CACTCTATGCCACGGTACACCAT
*RIG-I*	Forward Reverse	TTCAACTCCCAGTGTATGAGCAGC TGATGGAATTGTCCCATTGGTAAG
*IFI44L*	Forward Reverse	TAGGATAGCAGGAGCCACA TACGGATTTCTGAAACCAAGT
*OAS2*	Forward Reverse	TCCGCCATTCGGCTACAAAG CCTGGGAGCCTTCCATTTTG
*MX1*	Forward Reverse	GGCGTGGGAATCAGTCATG AGGAAGGTCTATGAGGGTCAGA
*ISG15*	Forward Reverse	GATCGGTGTGCCTGCCTTC CGTTGCTGCGACCCTTGT
*ISG56*	Forward Reverse	AAATGAATGAAGCCCTGGAGTATT AGGGATCAAGTCCCACAGATTTT

### Transfection and Reporter Gene Assays

Lipofectamine 2000 Transfection Reagents (Thermo Fisher Scientific) were used in all transfection assays following the manufacturer's protocols. As for the dual luciferase reporter assay, the monolayer cells were transfected with 0.1 μg of vector plasmids or the plasmids expressing various innate immune pathway components, and 0.1 μg of porcine IFN-β–promoter-luciferase reporter plasmid together with 0.01 μg of internal control pRL-TK plasmids for 24 h. The cells were lysed with passive lysis buffer, and the luciferase activities were determined using a dual-luciferase reporter assay kit (Promega) according to the manufacturer's instruction. All experiments were performed at least three times with three replicates each time. The data represent the means ± standard error of the results of three independent experiments.

### 50% Tissue Culture Infective Dose Assay

The 50% tissue culture infective dose (TCID_50_) titration method was used to determine viral titers. IBRS-2 or PK-15 cells were grown as monolayers in 96-well cell culture plates. A series of 10-fold dilutions of SVV or FMDV was used to infect the cells with eight replicates for each dilution. The incubated cells were cultured at a humidified incubator in 5% CO_2_ at 37 °C for 1 h. The supernatants were then removed, and the cells were washed with PBS for three times to detach the unabsorbed viruses. The cells were then maintained a low-serum media (DMEM containing 1% FBS) for 3 days. For the control, the cells were incubated with serum-free DMEM instead of the virus, and the experiment was carried out in parallel. The TCID_50_ values were calculated using the Reed–Muench method (37).

### Statistical Analysis

All results were presented as the means ± standard error. The significance was analyzed using GraphPad Prism (version 5.0; GraphPad Software, Inc) software. A Student's *t* test was employed for comparison of the differences between different groups. Statistical significance was defined as a *p* value of less than 0.05. Differences were considered to be statistically significant when the *p* value (∗) was less than 0.05 and to be highly significant when the *p* value (∗∗) was less than 0.01; ns indicated not significant.

## Results

### Quantitative Proteome Analysis of PK-15 and IBRS-2 Cells Infected by SVV

**Fig. 1:**
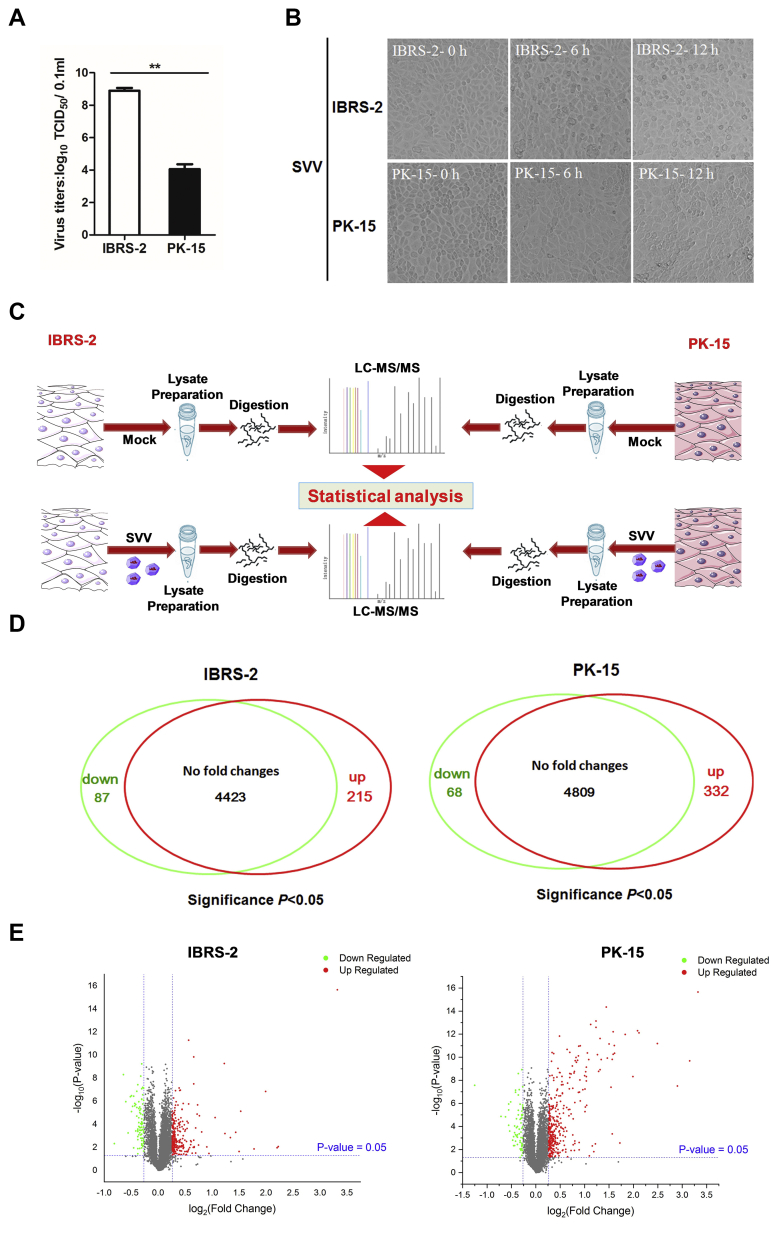
**Experimental design and comparison of the IBRS-2 and PK-15 cellular proteomes in response to SVV infection.***A*, IBRS-2 and PK-15 cells were infected with equal amounts of SVV, respectively, and the viral titers were determined by TCID_50_ assay. *B*, IBRS-2 cells or PK-15 cells were infected with 0.5 MOI of SVV for 0, 6, or 12 h. The virus-induced CPE was observed and recorded by the Olympus IX71 microscope. *C*, schematic illustration of the sample preparation. IBRS-2 and PK-15 cells mock infected or infected by SVV at an MOI of 0.5 for 6 h was subjected to iTRAQ-based proteomic analysis. *D*, the Venn diagram represents the number of DEPs identified in IBRS-2 and PK-15 cells in response to SVV infection. *E*, volcano plot comparing the fold-change difference of SVV-infected and mock-infected cells and the statistical significance of the observed differences. CPE, cytopathic effect; DEP, differentially expressed protein; IBRS-2, Instituto Biologico-Rim Suino-2; iTRAQ, isobaric tag for relative and absolute quantitation; MOI, multiplicity of infection; PK-15, porcine kidney-15 cells; SVV, Seneca Valley virus; TCID_50_, 50% tissue culture infective dose.

Both IBRS-2 and PK-15 cells are PK-derived epithelial cells, which have been widely used as models for studying the pathogenic mechanism of several porcine viral infectious diseases. Our previous study found that SVV multiplies much more efficiently in IBRS-2 cells than that in PK-15 cells. In the present study, we confirmed that SVV had higher titers in IBRS-2 cells than that in PK-15 cells as well (Fig. 1*A*). To elucidate the reasons behind the different manifestation, an unbiased high-throughput quantitative proteomic analysis of the landscape of IBRS-2 and PK-15 cells in response to SVV infection was performed. Equal amounts of IBRS-2 and PK-15 cells were infected by 0.5 MOI of SVV, respectively, and the CPEs were recorded and compared. The minimal CPE became visible approximately at 6 hpi in both IBRS-2 cells and PK-15 cells after SVV infection (Fig. 1*B*). Therefore, the proteome-wide analysis of the DEPs in IBRS-2 and PK-15 cells infected by SVV at 6 hpi was carried out by MS. The mock-infected or SVV-infected cells were collected and lysed and subjected to iTRAQ-based proteomic analysis (Fig. 1*C*). About 4725 and 5209 proteins were identified and quantified in IBRS-2 and PK-15 cells, respectively, after a stringent quality check and filtering of the data (FDR ≤ 0.01) (supplemental Table S1). Among these proteins, 215 upregulated DEPs and 87 downregulated DEPs were determined in IBRS-2 cells (supplemental Table S2), and 332 upregulated DEPs as well as 68 downregulated DEPs were identified in PK-15 cells (supplemental Table S3) during SVV infection (Fig. 1*D*), when setting the fold change >1.2 and the *p* value <0.05 (Fig. 1*E*). Raw data of MS have been deposited to the ProteomeXchange Consortium *via* the PRIDE with the dataset identifier PXD024002 and PXD024003, and the annotated spectra data (PRIDEXML file) can be viewed by PRIDE Inspector.

### Bioinformatics Analysis of the DEPs in SVV-Infected PK-15 and IBRS-2 Cells

**Fig. 2:**
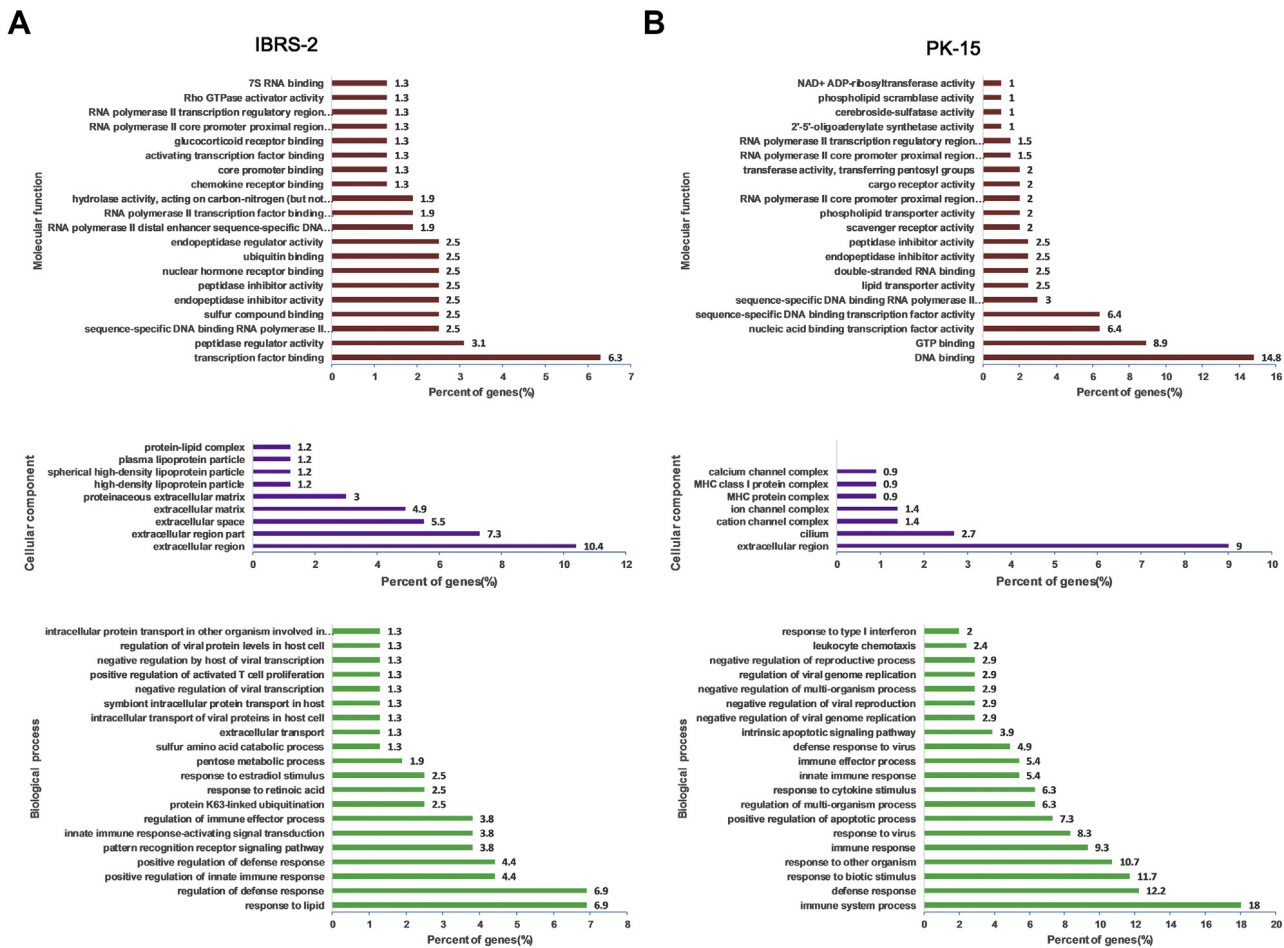
**GO enrichment analysis of the DEPs in SVV-infected IBRS-2 and PK-15 cells.***A*, the enrichment information of molecular function, cellular component, and biological process in IBRS-2 cells in response to SVV infection. *B*, the enrichment information of molecular function, cellular component, and biological process in PK-15 cells in response to SVV infection. DEP, differentially expressed protein; GO, Gene Ontology; IBRS-2, Instituto Biologico-Rim Suino-2; PK-15, porcine kidney-15 cells; SVV, Seneca Valley virus.

**Fig. 3:**
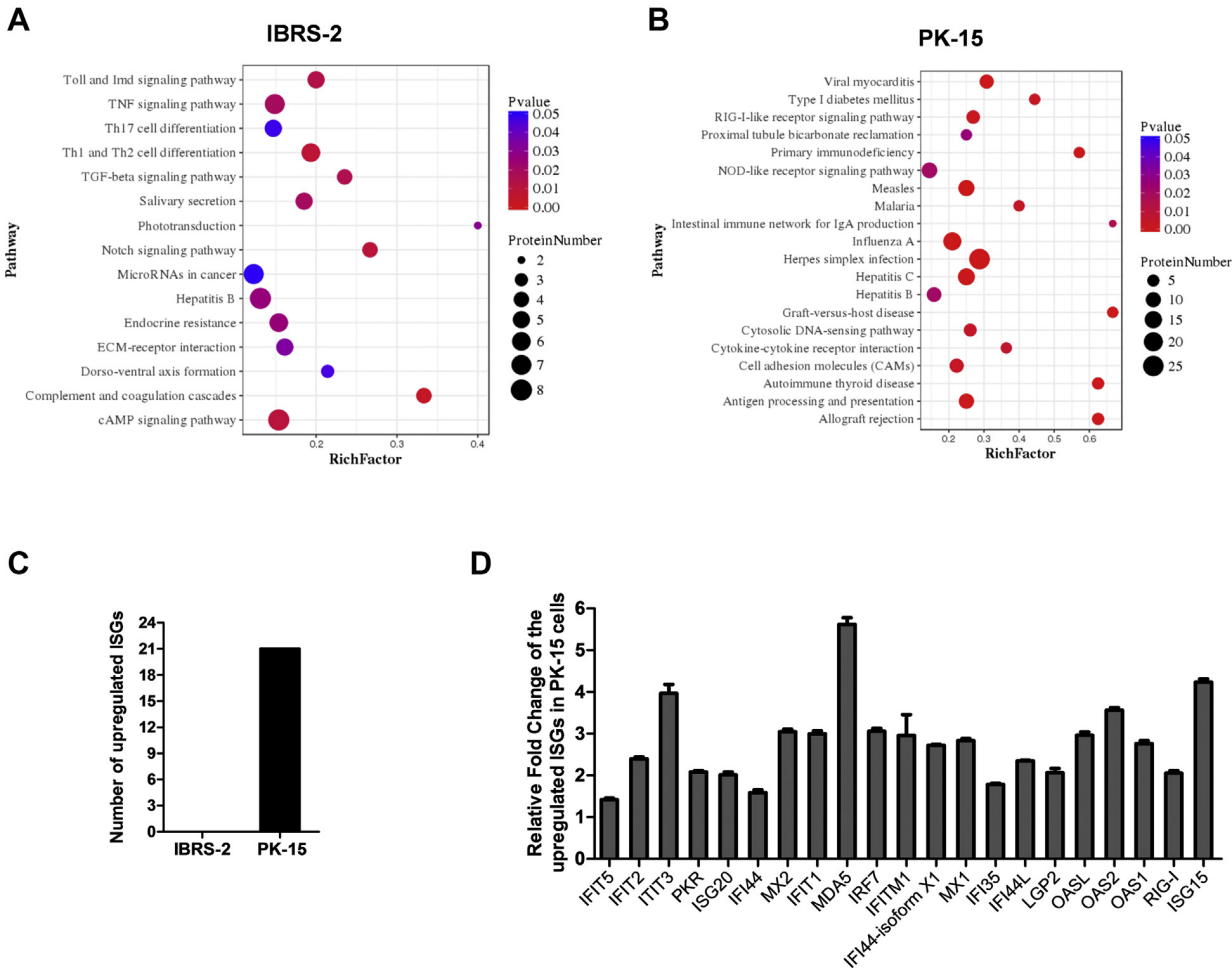
**Different innate immune responses in IBRS-2 and PK-15 cells in response to SVV infection.***A*, the KEGG pathway enrichment analysis for the DEPs in IBRS-2 cells in response to SVV infection (*p* value < 0.05). *B*, the KEGG pathway enrichment analysis for the DEPs in PK-15 cells in response to SVV infection (top 20 pathways). *C*, the number of differentially expressed ISGs identified in IBRS-2 and PK-15 cells in response to SVV infection. *D*, the 21 upregulated ISGs identified in SVV-infected PK-15 cells when setting the fold change >1.2 and the *p* value <0.05. DEP, differentially expressed protein; IBRS-2, Instituto Biologico-Rim Suino-2; ISG, interferon-stimulated gene; KEGG, Kyoto Encyclopedia of Genes and Genomes; PK-15, porcine kidney-15 cells; SVV, Seneca Valley virus.

The DEPs were searched against GO database to obtain enrichment information of molecular function, cellular component, and biological process. The top 20 most significant enrichment terms were extracted (*p* < 0.05) and shown in Figure 2 with the exception of the cellular component enrichment terms (the identified terms from the component ontology with *p* < 0.05 were less than 20). In IBRS-2 cells, the molecular functions of DEPs were mainly involved in transcription binding (6.3%), peptidase regulatory activity (3.1%), and sequence-specific DNA-binding RNA polymerase II transcription factor activity (2.5%). Many of the DEPs were the component of extracellular region (10.4%). Besides, many of the DEPs were enriched in response to lipid (6.9%) and regulation of defense response process (6.9%) (Fig. 2*A* and supplemental Table S4). In PK-15 cells, the molecular functions of DEPs were mainly involved in DNA binding (14.8%), GTP binding (8.9%), and nucleic acid–binding transcription factor activity (6.4%). Many of the DEPs were the component of extracellular region as well (9%). In addition, a large number of the DEPs were enriched in immune system process (18%) and defense response process (12.2%) (Fig. 2*B* and supplemental Table S5). The KEGG pathway enrichment analysis for the DEPs in IBRS-2 or PK-15 cells in response to SVV infection was performed, respectively. The enriched KEGG pathways at *p* value cutoff of 0.05 were obtained. The DEPs were mainly enriched in cAMP signaling pathway, complement and coagulation cascade pathway, and Notch signaling pathway in IBRS-2 cells (Fig. 3*A*). However, a large amount of DEPs were enriched in virus infection–related pathways (such as influenza A virus, herpes simplex infection, hepatitis C, and hepatitis B) in PK-15 cells. Meanwhile, many DEPs were enriched in the innate immune response–related pathways including RIG-I–like receptor signaling pathway, NOD-like receptor signaling pathway, and cytosolic DNA-sensing pathway (Fig. 3*B*).

The top 25 upregulated and downregulated DEPs in IBRS-2 cells and PK-15 cells in response to SVV infection were first extracted and compared. We found that there were 13 ISGs upregulated in SVV-infected PK-15 cells (Table 2). However, there was no upregulated ISGs in IBRS-2 cells (Table 3). Meanwhile, no ISGs were found in the downregulated DEPs in PK-15 (Table 4) and IBRS-2 (Table 5) cells. Therefore, we speculated that the highly expressed ISGs might have contributed to the decreased replication of SVV in PK-15 cells than that in IBRS-2 cells. We subsequently analyzed and compared all the DEPs identified in PK-15 cells and the DEPs in IBRS-2 cells. There were 21 ISGs upregulated in PK-15 cells after SVV infection (Fig. 3*C* and supplemental Table S3). In contrast, no ISGs were upregulated or downregulated in IBRS-2 cells after SVV infection (Fig. 3*C* and supplemental Table S2). The upregulated ISGs in PK-15 cells after SVV infection were shown in Figure 3*D*. All these ISGs have been reported to function as important antiviral proteins (38). These data implied that the aberrant IFN response in IBRS-2 cells during SVV infection might be responsible for the different viral titers observed in the two cell lines.

**Table 2:** Top 25 upregulated proteins induced by SVV infection in PK-15 cells

Accession no.	Protein	Fold change	*p*	Functions
gi|311271633	KIF11	10	2.20E-16	Microtubule motor activity
gi|350592923	Lipn	8.89	2.06E-10	Hydrolase activity
gi|37039803	env	7.47	3.14E-08	Unknown
gi|154147577	MDA5	5.62	6.54E-12	RNA helicase activity
gi|47523214	RSAD2	4.33	7.75E-13	Metal ion binding activity
gi|545892076	ISG15	4.24	4.99E-13	Ubiquitin protein ligase binding
gi|324123893	IFIT3	3.97	4.75E-09	RNA-binding activity
gi|72535208	OAS2	3.56	1.05E-12	2′-5′-oligoadenylate synthetase activity
gi|965785153	TTC40	3.3	0.002734	Cilium movement
gi|112790143	IRF7	3.06	4.51E-11	DNA-binding transcription factor activity
gi|206557741	MX2	3.05	9.69E-12	GTPase activity
gi|346986269	IFIT1	3	5.54E-11	Inhibiting expression of viral messenger RNAs
gi|350597057	TRIM5	3	6.17E-13	Ubiquitin–protein transferase activity
gi|72535194	OASL	2.96	1.53E-10	Double-stranded RNA–binding activity
gi|311250760	IFITM1	2.96	0.000443	Preventing viral fusion
gi|350588476	CASP13	2.91	4.00E-08	Cysteine-type endopeptidase activity
gi|545867461	MX1	2.83	7.06E-12	GTPase activity
gi|822599445	OAS1	2.76	1.19E-10	2′-5′-oligoadenylate synthetase activity
gi|545835544	IFI44-isoform X1	2.72	4.46E-15	Carbohydrate derivative–binding activity
gi|744614199	MCL1	2.53	1.58E-10	Protein transmembrane transporter activity
gi|311273188	SP140	2.49	1.19E-09	DNA-binding transcription factor activity
gi|350539097	UBE2L6	2.49	2.38E-12	Ubiquitin–protein transferase activity
gi|744587828	PRODH	2.44	1.68E-09	Proline dehydrogenase activity
gi|395147196	IFIT2	2.4	6.00E-11	Inhibiting expression of viral messenger RNAs
gi|594676074	LGALS3BP	2.35	7.20E-14	Scavenger receptor activity

**Table 3:** Top 25 upregulated proteins induced by SVV infection in IBRS-2 cells

Accession no.	Protein	Fold change	*p*	Functions
gi|335287990	OR6C2	10	2.20E-16	Olfactory receptor activity
gi|398314160	TRIB1	4.68	0.008714	Transferase activity
gi|965785153	TTC40	4.61	0.01107	Unknown
gi|440896262	TRIM34	3.98	1.44E-07	Protein kinase binding
gi|927211283	BARD1	3.43	0.01393	E3 ubiquitin transferase activity
gi|42764683	DUSP6	2.89	7.53E-06	Phosphoprotein phosphatase activity
gi|350587906	Arhgef38	2.83	0.02321	Guanyl-nucleotide exchange factor activity
gi|281350236	S100A2	2.71	0.000505	Calcium ion binding
gi|927218957	TEX11	2.53	0.001443	Initiation and/or maintenance of chromosome synapsis and formation of crossovers
gi|311247589	CCDC86	2.36	0.000648	RNA-binding activity
gi|347446703	TEX9	2.34	5.45E-10	Unknown
gi|311271633	KIF11	2.08	2.66E-05	Microtubule motor activity
gi|927123253	NOTCH2	1.92	0.0343	Calcium ion–binding activity
gi|567755274	MRPL14	1.87	0.009353	Structural constituent of ribosome
gi|927132603	ZNF384	1.75	0.000473	Nucleic acid–binding activity
gi|4503475	EEF1A2	1.7	1.86E-05	Translation elongation factor activity
gi|350583696	FAM118A	1.68	0.006462	Unknown
gi|927143047	TSEN34	1.66	2.12E-06	tRNA–intron endonuclease activity
gi|38569755	Vim	1.65	1.33E-05	Cell attachment, migration, and regulation
gi|586556365	TOMM6	1.62	0.03151	Metabolism of proteins and mitophagy
gi|149041900	Rplp1	1.62	0.000212	Protein kinase activator activity
gi|147906270	XIAP	1.59	0.01011	Ubiquitin–protein transferase activity
gi|927206547	ADAMTS14	1.59	0.008917	Metalloendopeptidase activity
gi|984144209	TUBA1C	1.58	0.002435	GTPase activity
gi|634844015	NUDT3	1.58	3.19E-08	Endopolyphosphatase activity

**Table 4:** Top 25 downregulated proteins induced by SVV infection in PK-15 cells

Accession no.	Protein	Fold change	*p*	Functions
gi|927109764	MAP4K4	0.42	2.71E-08	Creatine kinase activity
gi|350578534	HERC1	0.61	1.36E-05	Ubiquitin–protein transferase activity
gi|311267396	GSDMA	0.65	1.46E-05	Phosphatidylserine-binding activity
gi|335301427	DEPDC5	0.68	3.73E-06	GTPase activator activity
gi|311258138	NDUFA3	0.68	7.90E-05	NADH dehydrogenase activity
gi|927130383	XPOT	0.68	1.14E-06	Ran GTPase binding activity
gi|927171020	BANK1	0.69	0.000252	Signaling receptor binding
gi|311273225	ALPI	0.7	7.51E-07	Alkaline phosphatase activity
gi|149063967	Pck2	0.72	0.001552	Phosphoenolpyruvate carboxykinase activity
gi|350592831	ZSWIM8	0.73	0.004609	Zinc ion–binding activity
gi|507652967	TXNDC9	0.73	0.000768	Cadherin-binding activity
gi|148231384	TMSB10	0.74	9.12E-05	Organization of the cytoskeleton
gi|927221859	NEB	0.74	0.004553	Actin filament binding
gi|545887817	KCNJ16	0.74	0.000348	Voltage-gated ion channel activity
gi|545854656	TPTE2	0.75	0.001898	Protein tyrosine phosphatase activity
gi|350593017	MGEA5	0.75	7.19E-08	Hyalurononglucosaminidase activity
gi|114326214	HN1	0.75	1.08E-07	Inhibiting AR-signaling pathway
gi|927174867	PRSS23	0.75	0.000521	Serine-type endopeptidase activity
gi|575471149	FAU	0.76	2.15E-05	Structural constituent of ribosome
gi|298160948	COX6A1	0.76	0.000674	Oxidoreductase activity
gi|594666899	TSPAN6	0.76	0.000127	Regulation of cell development, activation, growth, and motility
gi|335285169	VAMP8	0.77	0.000871	Chloride channel inhibitor activity
gi|504183227	PCCB	0.77	0.000532	Acetyl-CoA carboxylase activity
gi|927096865	ATP9B	0.77	0.003652	Magnesium ion binding
gi|927096791	ALDH1A3	0.77	0.000101	Retinal dehydrogenase activity

**Table 5:** Top 25 downregulated proteins induced by SVV infection in IBRS-2 cells

Accession no.	Protein	Fold change	*p*	Functions
gi|335281692	C11orf83	0.57	0.00479	Phosphatidic acid binding
gi|113205858	CST3	0.64	5.02E-09	Endopeptidase inhibitor activity
gi|507949063	COPB1	0.66	1.11E-06	Structural molecule activity
gi|927096295	CASC4	0.68	0.0005738	Golgi apparatus
gi|545855557	HS6ST3	0.7	4.09E-07	Sulfotransferase activity
gi|47522832	IGFBP2	0.71	8.10E-06	Insulin-like growth factor–binding activity
gi|927109312	NOL8	0.71	3.35E-07	Regulation of RNA metabolic process
gi|284009806	ATP1A2	0.72	2.83E-06	Sodium:potassium-exchanging ATPase activity
gi|545849249	APLP2	0.73	3.72E-07	Serine-type endopeptidase inhibitor activity
gi|311275266	ADCK2	0.73	0.01134	Protein kinase activity
gi|77176666	B2M	0.74	1.59E-06	Protein homodimerization activity
gi|53987940	MORF4	0.74	2.56E-06	Unknown
gi|120564447	VTN	0.75	1.93E-06	Integrin binding activity
gi|335301929	OGDHL	0.75	0.0003839	Oxidoreductase activity
gi|545808910	AKAP8	0.76	3.54E-06	Anchoring protein activity
gi|940748915	HIST2H2AB	0.76	1.99E-05	Protein homodimerization activity
gi|311251198	CHCHD2	0.76	1.40E-07	Transcription factor binding
gi|507566291	RPLP1 isoform	0.77	0.0004215	Protein kinase activator activity
gi|594661345	PPP2R5C	0.77	0.01316	Peptidase activity
gi|343790888	FAM162A	0.77	0.03639	Regulation of apoptosis
gi|311266801	NUP85	0.77	3.76E-08	Nuclear pore complex assembly and maintenance
gi|545849414	PPP1R15B	0.77	0.0003452	Protein phosphatase regulator activity
gi|311271791	USMG5	0.77	0.0002639	Mitochondrial membrane ATP synthase
gi|927160558	OXA1L	0.77	0.00218	Membrane insertase activity
gi|927174867	PRSS23	0.77	0.001189	Serine-type endopeptidase activity

### Validation of DEPs by Western Blotting

**Fig. 4:**
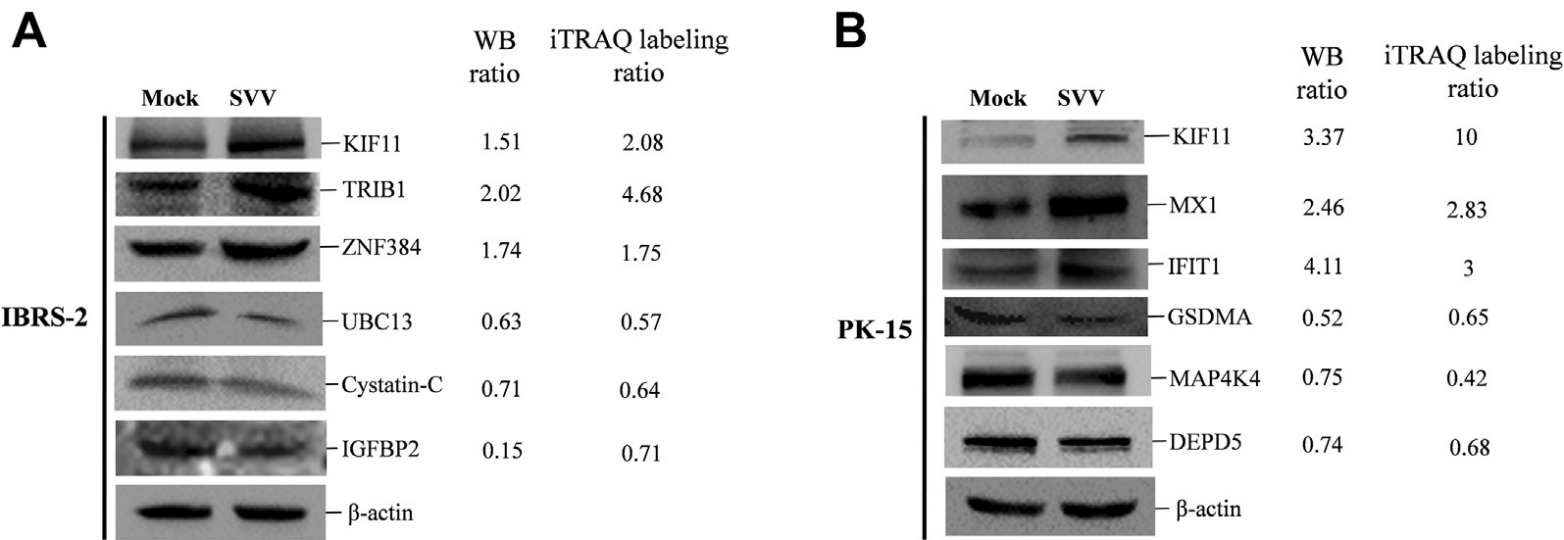
**Validation of DEPs by Western blotting (WB).***A*, IBRS-2 cells were infected with 0.5 MOI of SVV for 6 h. The expression of KIF11, TRIB1, ZNF384, UBC13, cystatin C, and IGFBP2 was determined by WB analysis. The relative fold change of the DEPs was determined by densitometric analysis after normalized to β-actin (WB ratio) and compared with the iTRAQ labeling ratio. *B*, PK-15 cells were infected with 0.5 MOI of SVV for 6 h. The expression of KIF11, MX1, IFIT1, GSDMA, MAP4K4, and DEPD5 was detected by WB. The WB ratio and iTRAQ labeling ratio were revealed and compared. DEP, differentially expressed protein; IBRS-2, Instituto Biologico-Rim Suino-2; MOI, multiplicity of infection; PK-15, porcine kidney-15 cells; SVV, Seneca Valley virus.

The quantitative proteomic results were verified by Western blotting analysis. Six DEPs in each cell line were selected to investigate the consistency of the iTRAQ and Western blotting analysis results, which showed that the expression trajectory of the six representative DEPs (KIF11, TRIB1, ZNF384, UBC13, cystatin C, and IGFBP2) in IBRS-2 cells in response to SVV infection was consistent with those determined by the iTRAQ analysis (Fig. 4*A*). Similarly, we analyzed the expression of six representative DEPs (KIF11, MX1, IFIT1, GSDMA, MAP4K4, and DEPD5) in PK-15 cells in response to SVV infection, and the Western blotting results were in agreement with the iTRAQ results as well (Fig. 4*B*). Overall, the Western blotting analysis verified changes of the identified DEPs by proteomic analysis of IBRS-2 and PK-15 cells in response to SVV infection.

### SVV Infection Induced Considerably Higher Levels of Type I IFN and ISGs in PK-15 Cells Than That in IBRS-2 Cells

**Fig. 5:**
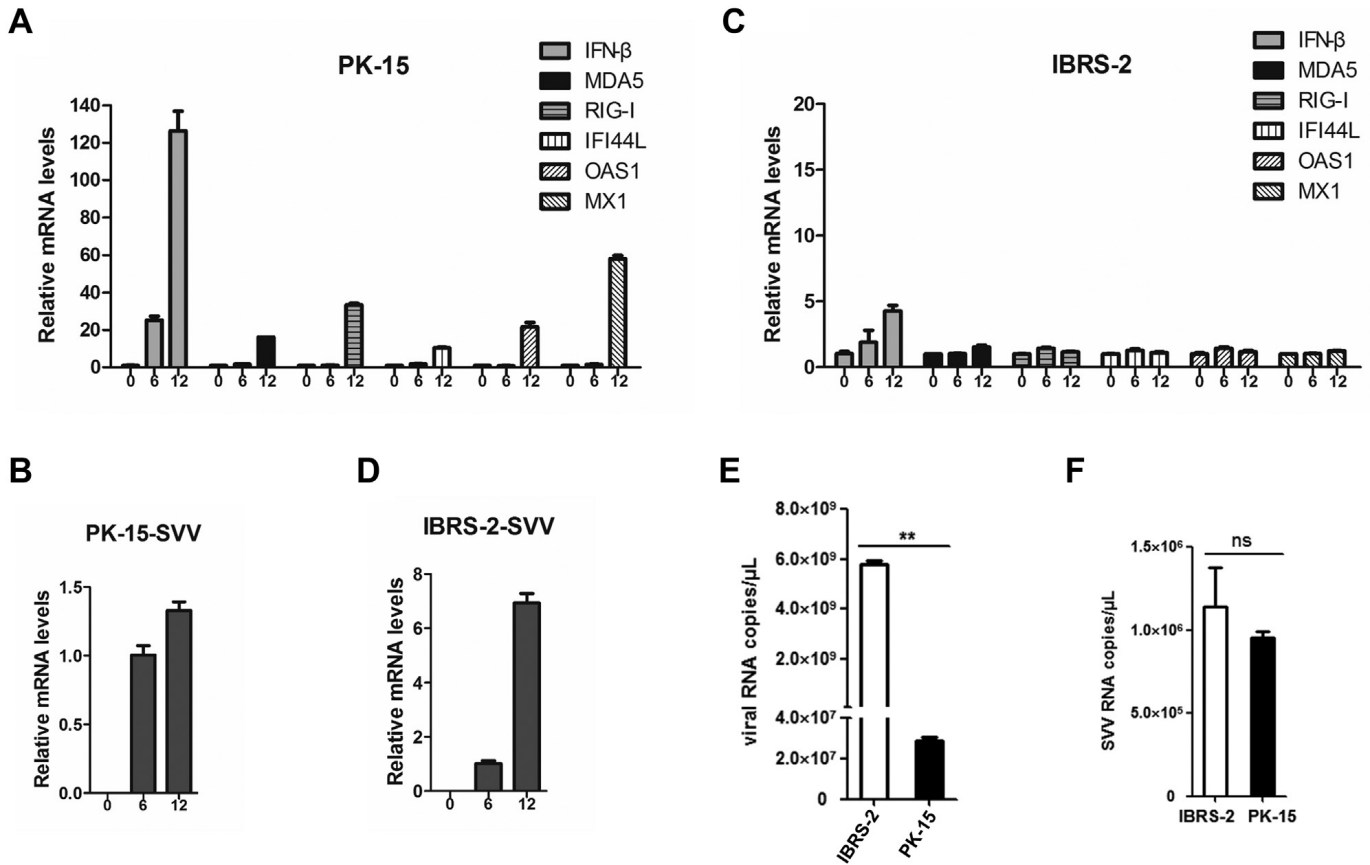
**Comparison of the IFN-β and ISG expression levels and viral loads in SVV-infected IBRS-2 and PK-15 cells.***A*, PK-15 cells were infected with 0.5 MOI of SVV for 0, 6, or 12 h. The expression level of IFN-β and MDA5, RIG-I, IFI44L, OAS1, as well as MX1 was measured by quantitative PCR (qPCR). *B*, the relative viral RNA abundance in PK-15 cells infected by SVV at 0, 6, and 12 hpi was determined by qPCR. *C*, IBRS-2 cells were infected with 0.5 MOI of SVV for 0, 6, or 12 h. The relative expression level of IFN-β and MDA5, RIG-I, IFI44L, OAS1, as well as MX1 was evaluated by qPCR. *D*, the relative expression level of viral RNA in IBRS-2 cells infected by SVV at 0, 6, and 12 hpi was determined by qPCR. *E*, the viral RNA copies in SVV-infected IBRS-2 and PK-15 cells at 12 hpi were determined and calculated. *F*, the viral RNA copies in SVV-infected IBRS-2 and PK-15 cells at 2 hpi were determined and compared. IBRS-2, Instituto Biologico-Rim Suino-2; IFN-β, interferon beta; ISG, interferon-stimulated gene; MOI, multiplicity of infection; ns, not significant; PK-15, porcine kidney-15 cells; SVV, Seneca Valley virus.

The KEGG pathway enrichment analysis showed that many DEPs were enriched in the innate immune response–related pathways including RIG-I–like receptor signaling pathway, NOD-like receptor signaling pathway, and cytosolic DNA-sensing pathway in PK-15 cells but not IBRS-2 cells during SVV infection (Fig. 3, *A* and *B*). Meanwhile, the quantitative proteome analysis indicated that a large amount of ISGs were induced by SVV in PK-15 cells but not IBRS-2 cells (Fig. 3, *C* and *D*). Therefore, we evaluated and compared the expression of type I IFN and ISGs in PK-15 and IBRS-2 cells during SVV infection. PK-15 and IBRS-2 cells were infected with equal amounts of SVV (0.5 MOI) for 0, 6, or 12 h. The expression level of IFN-β and several representative antiviral ISGs was measured. SVV infection remarkably induced the expression of IFN-β and various ISGs, and the expression levels gradually increased as infection progressed (Fig. 5*A*). High levels of ISGs were detected at 12 hpi. Therefore, the viral RNA amounts only increased 1.2- to 1.4-fold at 12 hpi compared with that at 6 hpi (Fig. 5*B*). In contrast, an extremely low level of IFN-β was induced by SVV infection in IBRS-2 cells, and we did not observe significant increase of ISG expression (Fig. 5*C*). As expected, the viral RNA amounts rapidly increased up to 6.4- to 7.5-fold at 12 hpi compared with that at 6 hpi (Fig. 5*D*). The viral RNA copy numbers in SVV-infected IBRS-2 and PK-15 cells at 12 hpi were measured and compared as well, which showed that there were much more viral RNA copies in the IBRS-2 cells than that in the PK-15 cells (Fig. 5*E*). To ensure that similar amount of viruses have entered into IBRS-2 and PK-15 cells at the beginning of infection, the replicative status of SVV in IBRS-2 and PK-15 cells at 2 hpi was evaluated and compared. The results showed that there were almost similar viral RNA copies in the IBRS-2 and PK-15 cells at the early infection stage (Fig. 5*F*). These data suggested that the divergent IFN response contributed to the different replication rates of SVV in PK-15 and IBRS-2 cells.

### FMDV Infection Also Induced Higher Type I IFN and ISG Expression in PK-15 Cells Than That in IBRS-2 Cells

**Fig. 6:**
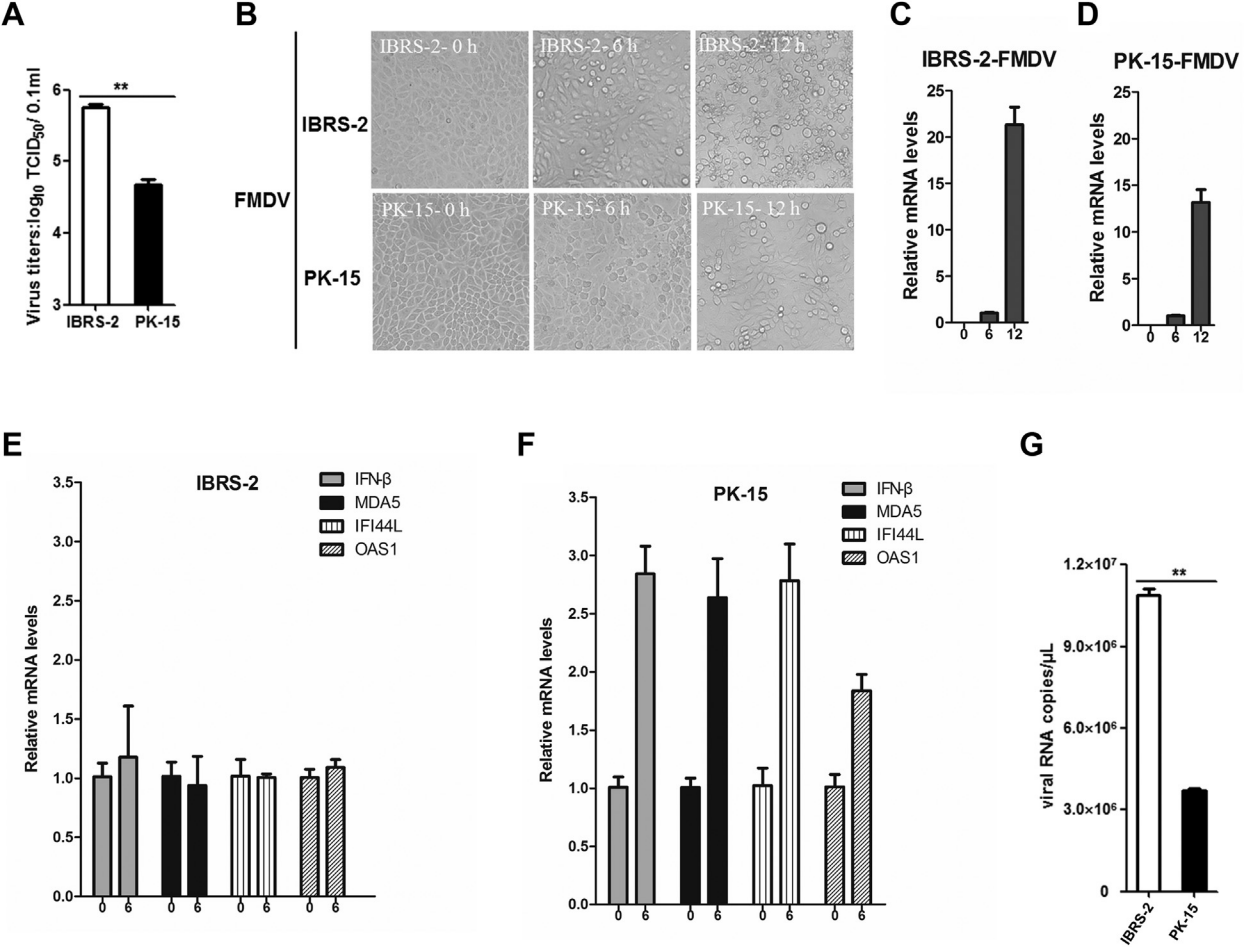
**The distinct type I IFN response and different replication rate of FMDV in IBRS-2 and PK-15 cells.***A*, IBRS-2 and PK-15 cells were infected with equal amounts of FMDV, respectively, and the viral titers were detected by TCID_50_ assay. *B*, IBRS-2 cells or PK-15 cells were infected with 0.1 MOI of FMDV for 0, 6, or 12 h. The virus-induced CPE was recorded by the Olympus IX71 microscope. *C*, the relative expression level of FMDV RNA in IBRS-2 cells at 0, 6, and 12 hpi was measured by quantitative PCR (qPCR). *D*, the relative expression level of FMDV RNA in PK-15 cells at 0, 6, and 12 hpi was determined by qPCR. *E*, the relative expression level of IFN-β, MDA5, IFI44L, and OAS1 in IBRS-2 cells infected by FMDV at 0 and 6 hpi was evaluated by qPCR. *F*, the relative expression level of IFN-β, MDA5, IFI44L, and OAS1 in PK-15 cells infected by SVV at 0 and 6 hpi was measured by qPCR. *G*, the viral RNA copies in FMDV-infected IBRS-2 and PK-15 cells at 6 hpi were determined and compared. CPE, cytopathic effect; FMDV, foot-and-mouth disease virus; IBRS-2, Instituto Biologico-Rim Suino-2; IFN, interferon; MOI, multiplicity of infection; PK-15, porcine kidney-15 cells; TCID_50_, 50% tissue culture infective dose.

We also evaluated the replication state of another porcine picornavirus FMDV in IBRS-2 and PK-15 cells. FMDV also had higher titers in IBRS-2 cells than that in PK-15 cells (Fig. 6*A*), and more significant CPE was observed in IBRS-2 cells as well (Fig. 6*B*). An approximately 21-fold viral RNA increase was detected in FMDV-infected IBRS-2 cells at 12 hpi compared with that at 6 hpi (Fig. 6*C*), whereas an approximately 13-fold change was determined in PK-15 cells (Fig. 6*D*). This suggested that FMDV replicated more rapidly in IBRS-2 cells than that in PK-15 cells. Severe CPE was caused by FMDV in IBRS-2 cells at 12 hpi, such as cell rounding and detachment (Fig. 6*B*). Therefore, the expression levels of IFN-β and several ISGs in FMDV-infected IBRS-2 and PK-15 cells at 6 hpi were measured, respectively. FMDV infection did not induce the increase of IFN-β and ISG expression in IBRS-2 cells (Fig. 6*E*), whereas it upregulated the expression of IFN-β and ISGs in PK-15 cells (Fig. 6*F*). As expected, more FMDV RNA copies were detected in IBRS-2 cells than that in PK-15 cells (Fig. 6*G*). These data indicated that the divergent IFN response also resulted in the different replication rate of FMDV in PK-15 and IBRS-2 cells.

### Type I IFN Signaling Pathways Were Intact in IBRS-2 Cells

**Fig. 7:**
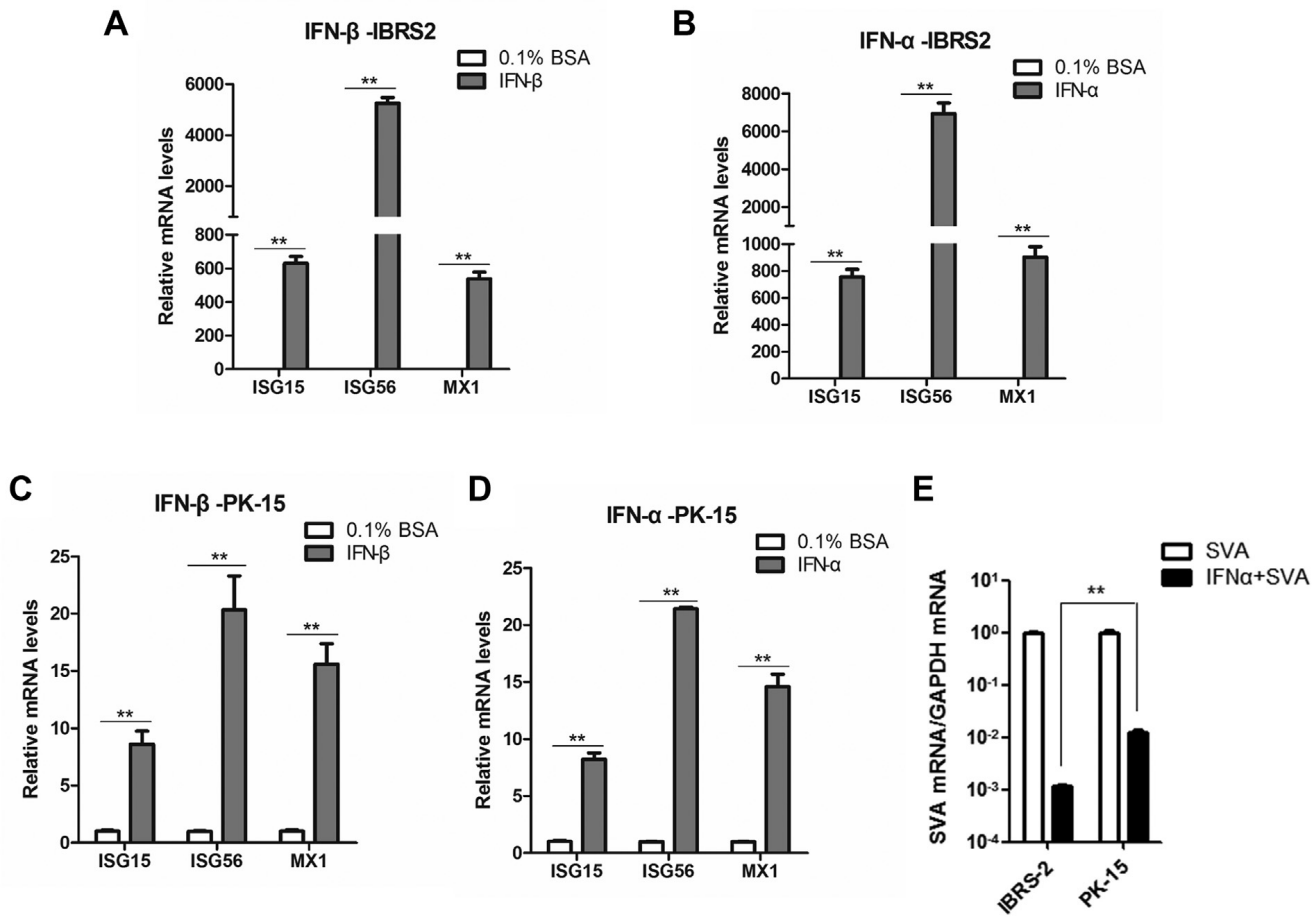
**The expression of ISGs in IBRS-2 and PK-15 cells in response to type I IFNs treatment.***A*, IBRS-2 cells were treated with porcine IFN-β or solvent control (0.1% BSA) for 12 h. The expression of ISG15, ISG56, and MX1 was measured by quantitative PCR (qPCR). *B*, IBRS-2 cells were treated with porcine IFN-α or solvent control (0.1% BSA) for 12 h. The expression of ISG15, ISG56, and MX1 was measured by qPCR. *C*, PK-15 cells were treated with porcine IFN-β or solvent control for 12 h. The expression of ISG15, ISG56, and MX1 was determined by qPCR. *D*, PK-15 cells were treated with porcine IFN-α or solvent control (0.1% BSA) for 12 h. The expression of ISG15, ISG56, and MX1 was measured by qPCR. *E*, IBRS-2 and PK-15 cells were treated with porcine IFN-α or 0.1% BSA for 12 h, respectively, and followed by SVA infection (0.5 MOI) for 12 h. The viral RNA levels were then measured and compared. BSA, bovine serum a lbumin; IBRS-2, Instituto Biologico-Rim Suino-2; IFN, interferon; ISG, interferon-stimulated gene; MOI, multiplicity of infection; PK-15, porcine kidney-15 cell; SVA, senecavirus A.

The expression of ISGs is regulated by IFNs through the Janus kinase signal transducer and activator of transcription (JAK-STAT) signaling pathway (39). To investigate whether the IBRS-2 cells refer to a dysfunction in JAK-STAT pathway signal transduction, IBRS-2 cells were treated with porcine IFN-β or protein control (0.1% bovine serum albumin) for 12 h. The mRNA expression level of three typical antiviral ISGs including ISG15, ISG56, and MX1 was measured. Incubation of porcine IFN-β notably induced the expression of these ISGs (Fig. 7*A*). We also determined that incubation of porcine IFN-α strikingly activated mRNA expression of ISG15, ISG56, and MX1 (Fig. 7*B*). As for PK-15 cells, incubation of porcine IFN-β or IFN-α could induce the mRNA expression of ISG15, ISG56, and MX1 as well (Fig. 7, *C* and *D*). These data suggested that the JAK-STAT pathway was functioning properly in IBRS-2 and PK-15 cells. Type I IFN signaling pathways were intact in IBRS-2 cells. Interestingly, we found that equal amount of type I IFN induced much higher expression of ISGs in IBRS-2 cells than that in PK-15 cells. To confirm these differences, same amount of IBRS-2 and PK-15 cells was treated with porcine IFN-α or 0.1% bovine serum albumin for 12 h and followed by senecavirus A infection for 12 h. The viral RNA was then measured and compared. SVV replication was decreased much more evident in IBRS-2 cells than that in PK-15 cells (Fig. 7*E*), confirming that the higher antiviral effect has been induced by IFN-α in IBRS-2 cells than that in PK-15 cells.

### TBK1-Mediated Signal Transduction Was Abnormal in IBRS-2 Cells

**Fig. 8:**
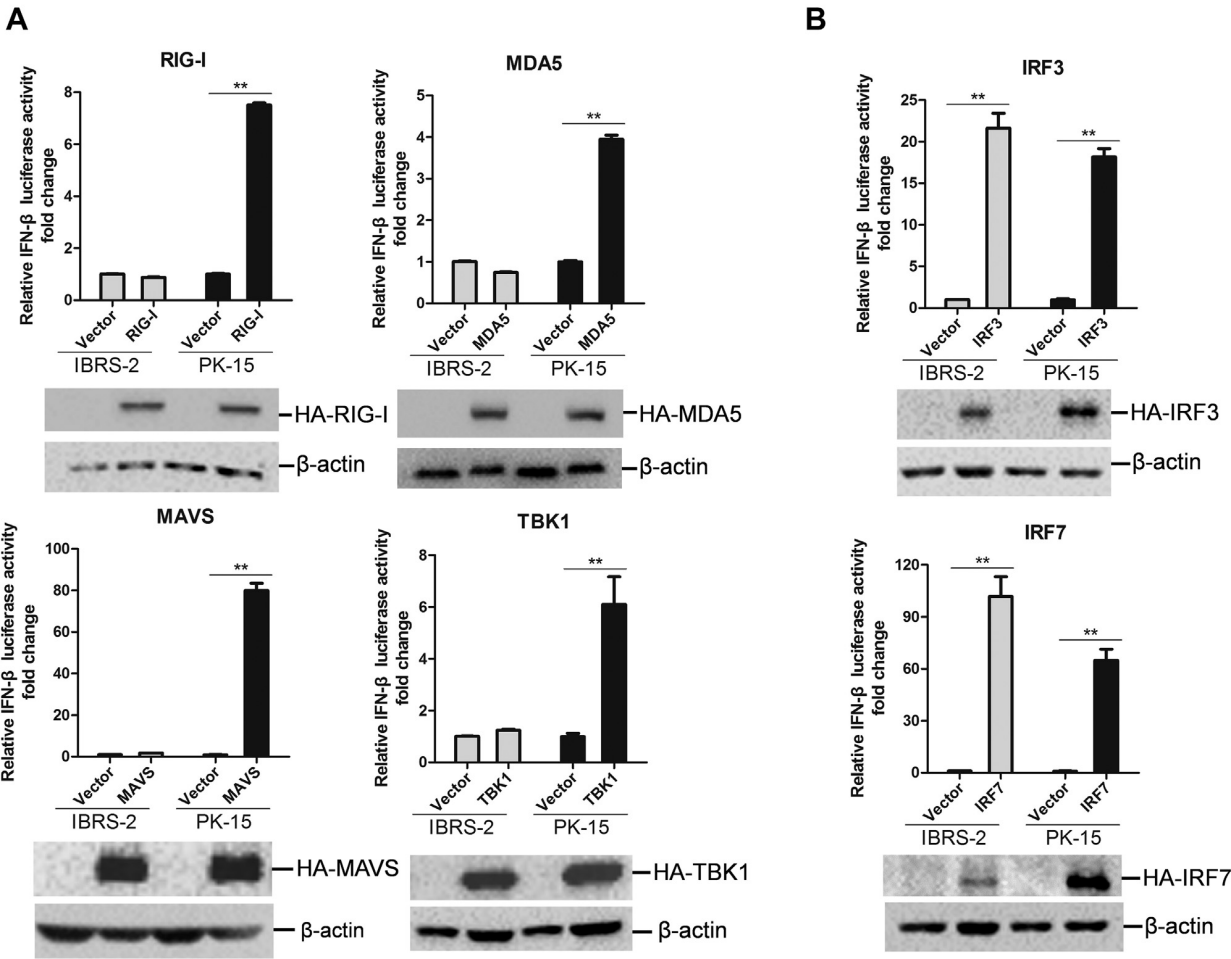
**The components of RLR signaling pathway-mediated IFN-β promoter activation in IBRS-2 and PK-15 cells.***A*, IBRS-2 cells or PK-15 cells were cotransfected with the plasmids expressing porcine RIG-I, MDA5, MAVS, or TBK1 together with the IFN-β promoter–driven luciferase reporter plasmids and the internal control pRL-TK plasmids. Luciferase activity was measured and analyzed at 24 h post-transfection. *B*, IBRS-2 cells or PK-15 cells were cotransfected with the plasmids expressing porcine IRF3 or IRF7 together with the IFN-β promoter–driven luciferase reporter plasmids and pRL-TK plasmids. Luciferase activity was measured and analyzed at 24 h post-transfection. The expression of these components was determined by Western blotting analysis. IBRS-2, Instituto Biologico-Rim Suino-2; IFN-β, interferon beta; IRF3, IFN regulatory factor 3; IRF7, IFN regulatory factor 7; PK-15, porcine kidney-15 cells; RLR, RIG-I–like receptor.

**Fig. 9:**
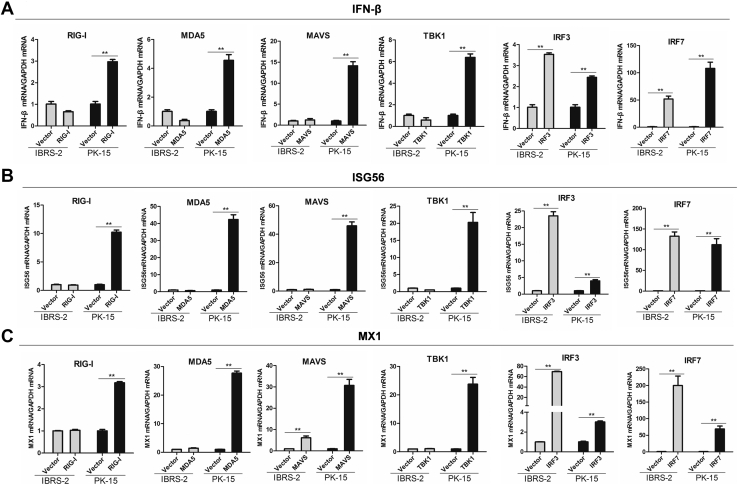
**The expression of IFN-β and ISGs in IBRS-2 and PK-15 cells induced by each component of RLR signaling pathway.** IBRS-2 cells or PK-15 cells were cotransfected with the plasmids expressing porcine RIG-I, MDA5, MAVS, TBK1, IRF3, or IRF7 for 24 h. The mRNA expression of IFN-β (*A*), ISG56 (*B*), and MX1 (*C*) was measured by quantitative PCR. IBRS-2, Instituto Biologico-Rim Suino-2; IFN-β, interferon beta; ISG, interferon-stimulated gene; PK-15, porcine kidney-15 cells; RLR, RIG-I–like receptor.

Given that both IBRS-2 cells and PK-15 cell line have a functioning JAK-STAT pathway, we speculated that the state of the RLR signaling pathway might be different between IBRS-2 and PK-15 cells. To analyze and determine the difference of the state of RLR signaling pathway between IBRS-2 and PK-15 cells, the activation of the IFN-β–promoter by a series of components of RLR signaling pathway was evaluated. We found that overexpression of RIG-I, MDA5, MAVS, or TBK1 only induced IFN-β–promoter activation in PK-15 cells but not in IBRS-2 cells (Fig. 8*A*). However, overexpression of IRF3 or IRF7 resulted in IFN-β–promoter activation both in PK-15 cells and IBRS-2 cells (Fig. 8*B*). As RIG-I, MDA5, and MAVS function upstream of TBK1, and both IRF3 and IRF7 function downstream of TBK1, this implied that the interface between TBK1 and IRF3/IRF7 was in a dysfunctional state. RIG-I-, MDA5-, MAVS-, TBK1-, IRF3-, or IRF7-induced mRNA expression of IFN-β, ISG56, and MX1 in IBRS-2 and PK-15 cells was further measured and compared. The results were consistent with the reporter assay data. Overexpression of RIG-I, MDA5, MAVS, or TBK1 was able to induce the expression of IFN-β in PK-15 cells but not in IBRS-2 cells, whereas overexpression of IRF3 or IRF7 remarkably upregulated IFN-β expression both in IBRS-2 and PK-15 cells (Fig. 9*A*). Overexpression of TBK1 also failed to induce the expression of ISG56 and MX1 in IBRS-2 cells (Fig. 9, *B* and *C*). These data suggested that TBK1-mediated signal transduction was abnormal in IBRS-2 cells.

**Fig. 10:**
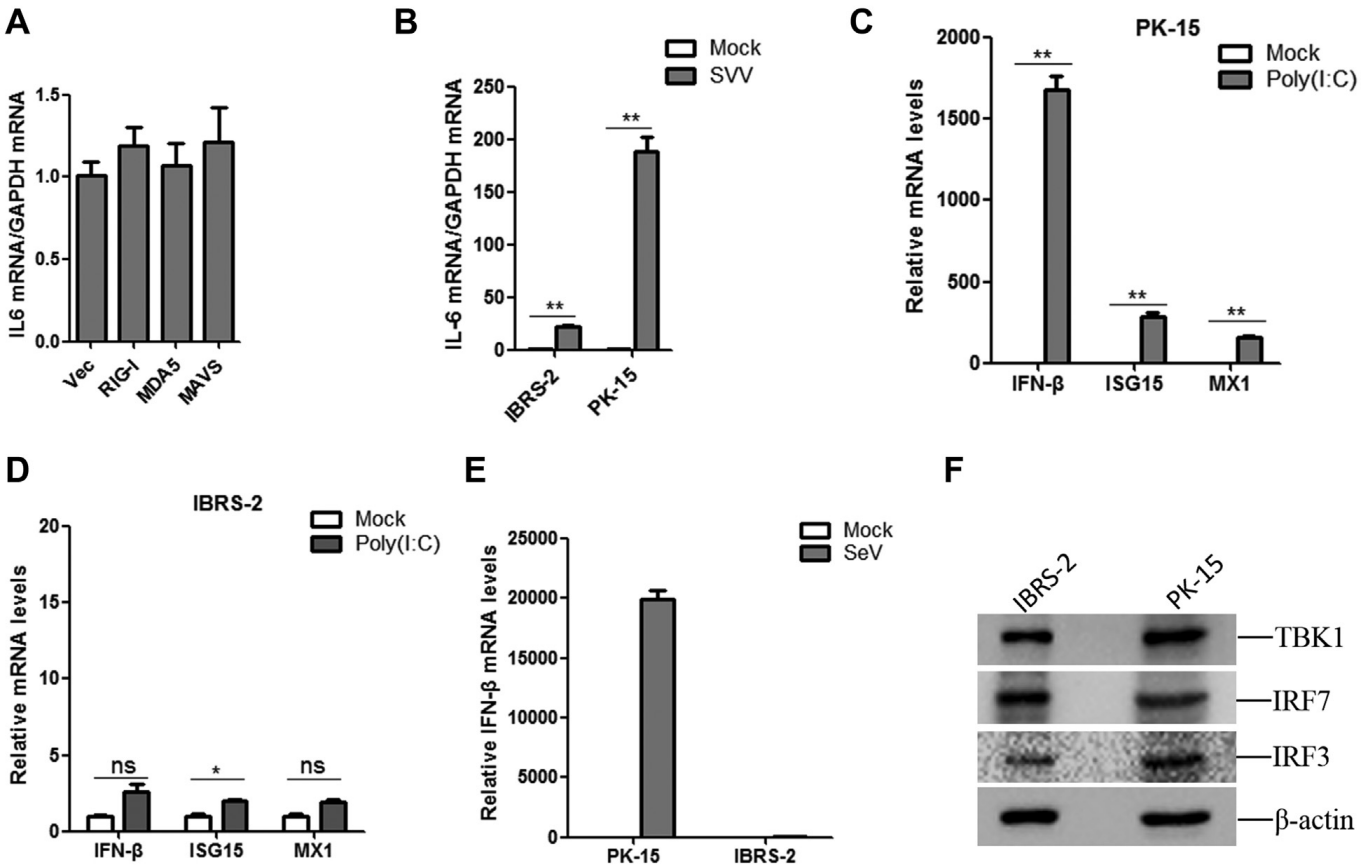
**The expression of IFN-β and ISGs in IBRS-2 and PK-15 cells induced by poly(I:C) and SeV.***A*, IBRS-2 cells were transfected with the vector plasmids or the plasmids expressing porcine RIG-I, MDA5, or MAVS for 24 h. The mRNA expression of IL-6 was measured by quantitative PCR (qPCR). *B*, IBRS-2 and PK-15 cells were infected with equal amounts of SVV for 12 h, respectively, and the mRNA expression of IL-6 was measured by qPCR. *C* and *D*, IBRS-2 and PK-15 cells were mock transfected or transfected with poly(I:C) for 12 h, respectively. The mRNA expression of IFN-β, ISG15, and MX1 was measured by qPCR. *E*, IBRS-2 and PK-15 cells were infected with equal amounts of SeV for 24 h, respectively, and the mRNA expression of IFN-β was measured by qPCR. *F*, equal amounts of IBRS-2 and PK-15 cells were collected and lysed with ice-cold lysis buffer. The protein levels of TBK1, IRF7, and IRF3 were measured by Western blotting analysis. IBRS-2, Instituto Biologico-Rim Suino-2; IFN-β, interferon beta; ISG, interferon-stimulated gene; IL-6, interleukin 6; IRF3, IFN regulatory factor 3; IRF7, IFN regulatory factor 7; PK-15, porcine kidney-15 cells; SeV, Sendai virus; SVV, Seneca Valley virus; TBK1, TANK-binding kinase 1.

The expression of interleukin-6 (IL-6) in RIG-I, MDA5, and MAVS overexpressing IBRS-2 cells was evaluated, respectively. Overexpression of RIG-I, MDA5, or MAVS did not induce the expression of IL-6 in IBRS-2 cells (Fig. 10*A*), suggesting that RIG-I, MDA5, and MAVS could not activate NF-κB pathway in IBRS-2 cells. This also implied the dysfunction of the RLR pathway in IBRS-2 cells. The expression of IL-6 in SVV-infected PK-15 and IBRS-2 cells was investigated as well. SVV infection induced the expression of IL-6 in both PK-15 and IBRS-2 cells. However, SVV infection induced extremely higher expression of IL-6 in PK-15 cells than that in IBRS-2 cells (Fig. 10*B*), suggesting a different signal transduction manner in the two cell lines.

To verify the abnormality of RLR pathway signal transduction in IBRS-2 cells under various stimulation, the viral RNA mimetic poly(I:C) was used to activate the RLR pathway, and the expression level of IFN-β and ISGs was measured in IBRS-2 and PK-15 cells, respectively. Transfection of poly(I:C) significantly induced the expression of IFN-β and ISGs in PK-15 cells (Fig. 10*C*). However, it only weakly induced IFN-β and ISG expression in IBRS-2 cells (Fig. 10*D*). Sendai virus (SeV) has been widely used as an RNA model virus to strongly induce type I IFN production (40). We also evaluated the expression level of IFN-β induced by SeV infection in IBRS-2 and PK-15 cells. Similarly, SeV infection strikingly induced the expression of IFN-β in PK-15 cells but not in IBRS-2 cells (Fig. 10*E*). All these data confirmed the abnormality of RLR pathway signal transduction in IBRS-2 cells. TBK1-mediated signal transduction was abnormal in IBRS-2 cells. The CDS of TBK1, IRF3, and IRF7 was analyzed and compared, and the sequencing results showed that TBK1, IRF3, and IRF7 showed 100% identity in PK-15 and IBRS-2 cells (data not shown). The expression level of TBK1, IRF3, and IRF7 was analyzed as well, which showed that the expression levels of TBK1, IRF3, and IRF7 were almost similar in PK-15 and IBRS-2 cells (Fig. 10*F*). These data further implied that the signal transduction from TBK1 to IRF3 in the RLR signaling pathway in IBRS-2 cells was dysfunctional.

## Discussion

Both IBRS-2 and PK-15 cells have been widely used for porcine picornavirus research (10, 41, 42, 43, 44, 45, 46). SVV replication in IBRS-2 cells is relatively fast, and the infection results in severe CPE (10). The replication of SVV in PK-15 cells appears to differ from that in IBRS-2 cells, and gentle CPE occurs in SVV-infected PK-15 cells. A better understanding of the underlying mechanism of the different cytopathic manifestation caused by SVV in IBRS-2 and PK-15 cells will be crucial for studying the pathogenesis of SVV and realizing the context and properties of the two cell lines. Using SVV as a case study, we determined the proteome landscape and cellular responses of IBRS-2 cells as well as PK-15 cells in response to viral infection. This was the first study to characterize the proteomic profile of SVV-infected porcine cells. We comprehensively compared the expression features between PK-15 cells and IBRS-2 cells after SVV infection. Based on the GO analyses, many of the proteins involved in transcription factor–binding function were regulated in IBRS-2 cells following SVV infection. We found one of the upregulated DEPs, WWP2, which is annotated to the term transcription factor–binding function, negatively regulates host innate immune response by targeting TRIF for ubiquitination and degradation (47). TRIF is an important adaptor protein in the TLR response to viral infection (48). The upregulation of WWP2 during SVV infection in IBRS-2 cells might have impaired the innate immune response. In addition, the biological process analysis revealed that a large amount of upregulated DEPs identified in PK-15 cells were enriched in immune system process (18%) and defense response process (12.2%). There were many ISGs found in these DEPs, which implied a stronger innate immune response in PK-15 cells during SVV infection. The KEGG pathway enrichment analysis also indicated that the innate immune response–related pathways (including RLR signaling pathway, NOD-like receptor signaling pathway, and cytosolic DNA-sensing pathway) were efficiently regulated in PK-15 cells but not in IBRS-2 cells during SVV infection. What is more, 13 ISGs were identified in the TOP 25 upregulated DEPs in PK-15 cells, and there was no upregulated ISGs in IBRS-2 cells. The cAMP signaling pathway, complement and coagulation cascade pathway, Notch signaling pathway and NOD-like receptor signaling pathway, and cytosolic DNA-sensing pathways were regulated by SVV. All these pathways are involved in modulation of immune responses. All these data suggested that the considerably divergent innate immune response triggered by SVV in IBRS-2 and PK-15 cells might have been responsible for the different cytopathic manifestation during the infection.

The innate immune response is the rapid defense response triggered by invading pathogens. The cellular factors that mediate this defense are the products of ISGs and various cytokines during the innate immune response. The pattern recognition receptors are responsible for sensing the microbial infections, initiating the innate immune response and induction of a number of antiviral cellular factors to eliminate or, at least, to contain infectious agents (49). RLRs are cytoplasmic pattern recognition receptors that are responsible for sensing RNA virus infections and modulating the expression of type I IFNs and other proinflammatory cytokines that collectively establish an antiviral state (50). SVV, as a porcine picornavirus, has a single-stranded genomic RNA, and our previous study determined that the RLR signaling pathway is in charge of induction of type I IFNs and ISG expression and establishment of antiviral state during SVV infection (51). In SVV-infected PK-15 cells, the proteomic analysis totally identified 21 upregulated ISGs. In contrast, there were no upregulated ISGs determined in SVV-infected IBRS-2 cells. We also verified this expression feature in the two cell lines through qPCR analysis. All these data indicated that the RLR signaling pathway-mediated IFN response is inactive during SVV infection in IBRS-2 cells.

During RNA virus infections, the RNA from viruses bind to RLRs after entry into the host cell and elicit activation of the members of RLRs (RIG-I or MDA5) (50). The activated RLRs recruit the adaptor molecule MAVS at the mitochondrial membrane to induce the formation of MAVS filaments. The serine/threonine protein kinase TBK1 then recruited to MAVS filaments leads to the assembly of a complex that is essential for activation of IRF3 and IRF7. The activated IRF3/IRF7 is imported into the nucleus and contributes to transcriptional activation of type I IFNs (52, 53, 54). The secreted type I IFNs bind to IFN receptors and initiate JAK-STAT signaling and the ISGF3-dependent expression of various ISGs (39). In the RLR pathway, TBK1 is also critical for the activation of transcriptional factor NF-κB that regulates the expression of various proinflammatory cytokines (such as IL-6 and interleukin-1β). In the present study, we found that SVV infection induced the expression of IL-6 in both PK-15 and IBRS-2 cells. However, SVV infection induced extremely higher expression of IL-6 in PK-15 cells than that in IBRS-2 cells. Although the RLR pathway is abnormal in IBRS-2 cells, the TNF-α–mediated NF-κB pathway might have been activated during SVV infection. This explained why SVV infection partly induced the expression of IL-6 in IBRS-2 cells. Overexpression of RIG-I, MDA5, or MAVS could not induce the expression of IL-6 in IBRS-2 cells, suggesting that RIG-I, MDA5, and MAVS failed to activate NF-κB pathway in IBRS-2 cells. This confirmed the dysfunction of the RLR pathway in IBRS-2 cells. Type I IFNs could efficiently induce ISG expression in IBRS-2 cells. This determined that type I IFN signaling pathways were intact in IBRS-2 cells. Furthermore, equal amount of IFN-β or IFN-α even induced much higher expression of ISGs in IBRS-2 cells than that in PK-15 cells. More extensive studies are required to determine the differences of the signal transduction process initiated by type I IFNs in the two cell lines.

After confirmation of having intact type I IFN signaling pathways in IBRS-2 cells, we explored the state of RLR pathway in IBRS-2 cells and determined that TBK1-mediated signal transduction was abnormal in IBRS-2 cells. The interface between TBK1 and IRF3 or IRF7 was in a dysfunctional state. TBK1 is a serine/threonine protein kinase involved in many signaling pathways and forms a node between them (55). TBK1 plays an important role in RLR pathway. The transcription factors IRF3 and IRF7 are required for the expression type I IFNs and many genes involved in the innate immune response (56). TBK1 is an extremely critical physiological kinase that mediates phosphorylation and activation of IRF3/IRF7 (57, 58). TBK1 phosphorylates IRF3 at the Ser-386 and results in its activation and induces type I IFNs (59). Overexpression of TBK1 failed to induce IFN-β and ISG expression, whereas overexpression of IRF3/IRF7 efficiently motivated the expression of IFN-β and ISG expression in IBRS-2 cells. This implies that the endogenous IRF3 and IRF7 might not be working normally or properly in IBRS-2 cells. The CDS sequences and expression levels of IRF3 and IRF7 were similar in PK-15 and IBRS-2 cells. The crucial phosphorylation sites in IRF3 and IRF7 targeted by TBK1 was same. This suggested that IRF3 and IRF7 were not responsible for the aberrant signal transduction in IBRS-2 cells. The abnormal signal transduction from TBK1 to IRF3 and IRF7 has contributed to the different innate immune response in the two cell lines. The reaction between TBK1 and IRF3 or IRF7 should be further exploited. Besides, the E3 ligase WWP2 negatively regulates TLR3-mediated innate immune response (47). WWP2 was upregulated in IBRS-2 cells during SVV infection, whether this is another factor to impair host innate immune response in IBRS-2 cells remains unknown.

The kidney is one of the reservoirs of SVV in pigs (60, 61). Investigation of the replication state of SVV in PK cells is helpful for clarification of the pathogenesis of SVV. In addition, the significance of cell line infection studies is involved in the oncolytic virotherapy research. SVV is an oncolytic virus, and it has been used as an oncolytic virotherapy candidate in humans (1, 2). SVV has been shown to reveal strong cytotoxic effect on small-cell lung cancer cell lines and solid pediatric cancer cell lines (1). It is reported that SVV plays an effective role to suppress invasive and metastatic retinoblastoma (62). Phase II study of SVV in patients with small-cell lung cancer has been carried out. However, the rapid clearance of the virus by host is still a problem that hinders the development of clinical trials (63). SVV can replicate in normal swine kidney cells in pigs (60, 61). The pig is very similar to humans with regard to anatomy, genetics, and physiology (20). Therefore, pig could be used as a more prominent model for study of the oncolytic virotherapy of SVV (such as renal cell carcinoma). Our data in this study suggested that blocking the RLR pathway signal transduction in SVV-infected cells will efficiently promote SVV replication. IBRS-2 cells supply a more favorable environment for SVV replication, but which protein has contributed to this phenotype remains unknown. The underlying mechanisms will provide new potential targets and an insight for oncolytic virotherapy of SVV.

Although much more work is required in order to elucidate the mechanisms of aberrant RLR signaling in IBRS-2, our studies have uncovered a different characteristic between IBRS-2 and PK-15 cell line during porcine picornaviral infection. PK-15 cell line can be used as a model for evaluation of porcine picornavirus–induced innate immune signaling and the involved antagonistic effects caused by viral proteins. However, the IBRS-2 cell line is not applicable to investigate porcine picornavirus–induced innate immune signaling. What is more, IBRS-2 cell line has a significant advantage over PK-15 cell line in multiplying porcine picornaviruses, which is more properly for isolation of porcine picornaviruses. This provides useful guidance for choosing right cell line in porcine picornaviruses–mediated host response research and could be easily extended to other porcine viruses. More importantly, this will provide an insight for decreasing SVV clearance rate in cancer cells.

**Fig. 11:**
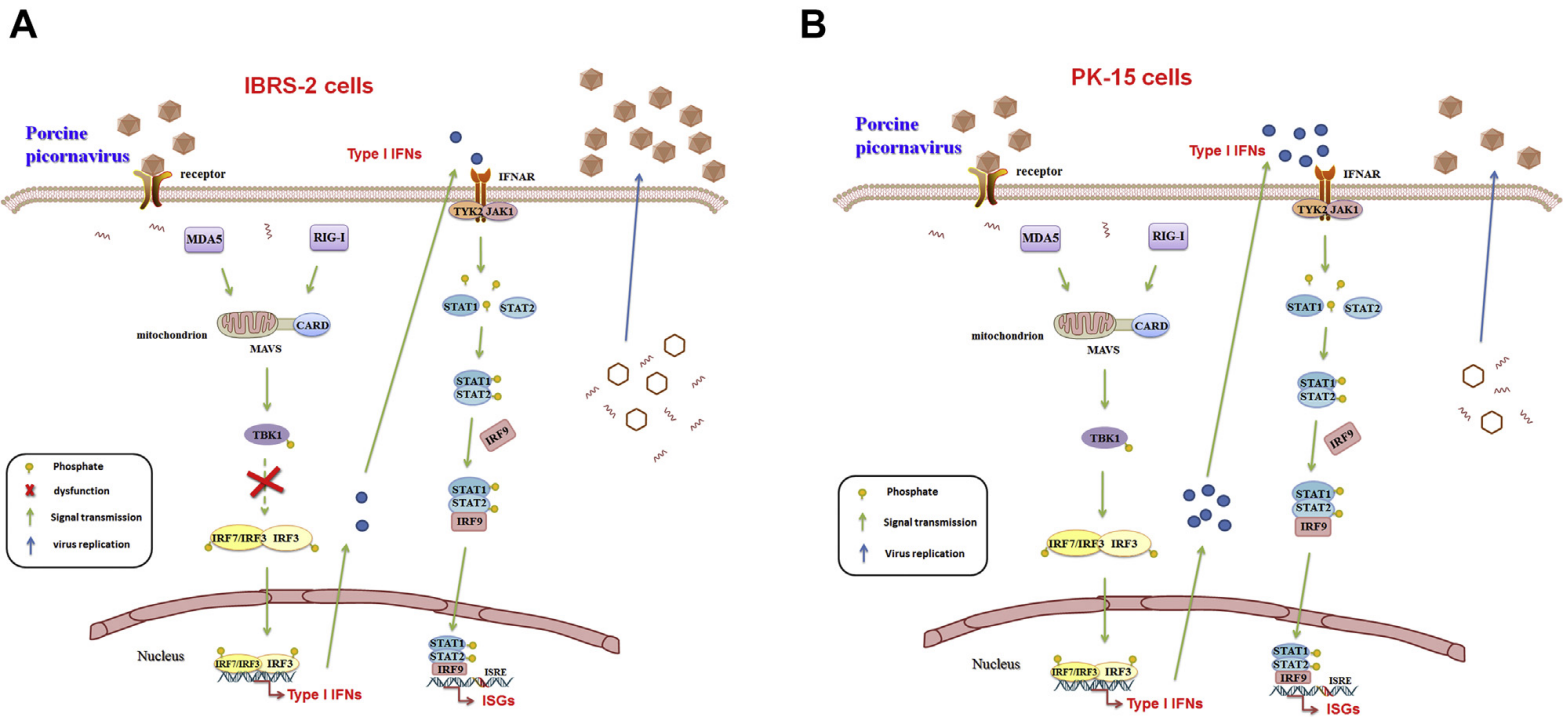
**Schematic overview of the RLR signaling pathway and JAK-STAT signaling pathway in IBRS-2 and PK-15 cell lines.***A*, the signal transduction from TBK1 to IRF3 in the RLR signaling pathway in IBRS-2 cells is dysfunctional, whereas the JAK-STAT signaling pathway is functioning properly during porcine picornavirus infection. The aberrant production of type I IFNs contributes to rapid replication of the virus. *B*, both the RLR signaling pathway and JAK-STAT signaling pathway function properly in PK-15 cells during porcine picornavirus infection, which resulted in a slower replication of the virus compared with that in IBRS-2 cells. IBRS-2, Instituto Biologico-Rim Suino-2; IFN, interferon; IRF3, IFN regulatory factor 3; JAK-STAT, Janus kinase signal transducer and activator of transcription; PK-15, porcine kidney-15 cells; RLR, RIG-I–like receptor; TBK1, TANK-binding kinase 1.

In summary, our study investigated the proteome landscape of SVV-infected IBRS-2 and PK-15 cells, respectively, by iTRAQ technology. We determined that porcine picornavirus infection could not induce sufficient activation of RLR pathway in IBRS-2 cells, while it highly activated RLR pathway in PK-15 cells. The aberrant signal transduction from TBK1 to IRF3 in IBRS-2 cells was the fundamental cause of the different innate immune response manifestation and viral replication rate in the two cell lines (Fig. 11). Further studies are required to determine the differences of the signal transduction process mediated by TBK1 in the two cell lines. The underlying mechanisms will provide new targets and an insight for decreasing the viral clearance rate of SVV and probably improve the oncolytic effect by SVV in cancer cells.

## Data Availability

The MS proteomics data have been deposited to the ProteomeXchange Consortium using the PRIDE partner repository (http://www.ebi.ac.uk/pride/archive/ with the dataset identifiers PXD024002 [for PK-15 cells] and PXD024003 [for IBRS-2 cells]).

## Supplemental data

This article contains supplemental data.

## Conflict of interest

The authors declare no competing interests.
